# CD45RO^-^CD8^+^ T cell-derived exosomes restrict estrogen-driven endometrial cancer development via the ERβ/miR-765/PLP2/Notch axis

**DOI:** 10.7150/thno.58337

**Published:** 2021-03-11

**Authors:** Wen-Jie Zhou, Jie Zhang, Feng Xie, Jiang-Nan Wu, Jiang-Feng Ye, Jian Wang, Ke Wu, Ming-Qing Li

**Affiliations:** 1 Laboratory for Reproductive Immunology, NHC Key Lab of Reproduction Regulation (Shanghai Institute of Planned Parenthood Research), Hospital of Obstetrics and Gynecology, Shanghai Medical School, Fudan University, Shanghai 200080, People's Republic of China.; 2 Reproductive Medical Center, Department of Obstetrics and Gynecology, Ruijin Hospital, Shanghai Jiao Tong University School of Medicine, Shanghai 200025, People's Republic of China.; 3 Department of Obstetrics and Gynecology, Shanghai General Hospital, Shanghai Jiao Tong University School of Medicine, No.100, Haining Road, Shanghai 200080, People's Republic of China.; 4 Clinical Epidemiology, Hospital of Obstetrics and Gynecology, Shanghai Medical School, Fudan University, Shanghai 200011, People's Republic of China.; 5 Division of Obstetrics and Gynecology, KK Women's and Children's Hospital, 229899, Singapore.; 6 Department of Urology, Shanghai General Hospital, Shanghai Jiao Tong University School of Medicine, No.100, Haining Road, Shanghai 200080, People's Republic of China.; 7 Shanghai Key Laboratory of Female Reproductive Endocrine Related Diseases, Hospital of Obstetrics and Gynecology, Shanghai Medical School, Fudan University, Shanghai, 200080, People's Republic of China.

**Keywords:** miR-765, PLP2, estrogen, T cell-derived exosomes, endometrial cancer

## Abstract

**Rationale:** Estrogen-dependent cancers (e.g., breast, endometrial, and ovarian cancers) are among the leading causes of morbidity and mortality in women worldwide. Recently, exosomes released by tumor-infiltrating CD8^+^ T cells have been under the spotlight in the field of cancer immunotherapy. Our study aims at elucidating the underlying mechanisms of the crosstalk between estrogen signaling and CD8^+^ T cells, and possible intervention values in uterine corpus endometrial cancer (UCEC).

**Methods:** Micro RNA-seq was conducted to screen differentially expressed micro RNA in UCEC. Bioinformatic analysis was processed to predict the target of miR-765. RNA silencing or overexpressing and pharmacologic inhibitors were used to assess the functions of ERβ/miR-765/PLP2/Notch axis in UCEC cell proliferation and invasion *in vivo* and *in vitro*. *In vivo* imaging was performed to evaluate the metastasis of tumor in mice. Combined fluorescent *in situ* hybridization for miR-765 and immunofluorescent labeling for CD8 was carried out to prove the co-localization between miR-765 and CD8^+^ T cells. Exosomes derived from CD45RO^-^CD8^+^ T cells were isolated to detect the regulatory effects on UCEC.

**Results:** miR-765 is characterized as the most downregulated miRNA in UCEC, and there is a negative correlation between miR-765 and Proteolipid protein 2 (PLP2) in UCEC lesion. Estrogen significantly down-regulates miR-765 level, and facilitates the development of UCEC by estrogen receptor (ER) β. Mechanistically, this process is mediated through the miRNAs (e.g., miR-3584-5p, miR-7-5p, miR-150-5p, and miR-124-3p) cluster-controlled regulation of the PLP2, which further regulates Ki-67 and multiple epithelial-mesenchymal transition (EMT)-related molecules (e.g, E-cadherin and Vimentin) in a Notch signaling pathway-dependent manner. Interestingly, the selective ER degrader Fulvestrant alleviates estrogen-mediated miR-765/PLP2 expression regulation and UCEC development in ERβ-dependent and -independent manners. Additionally, CD45RO^-^CD8^+^ T cell-derived exosomes release more miR-765 than that from CD45RO^+^CD8^+^ T cells. In therapeutic studies, these exosomes limit estrogen-driven disease development via regulation of the miR-765/PLP2 axis.

**Conclusions:** This observation reveals novel molecular mechanisms underlying estrogen signaling and CD8^+^ T cell-released exosomes in UCEC development, and provides a potential therapeutic strategy for UCEC patients with aberrant ERβ/miR-765/PLP2/Notch signaling axis.

## Introduction

As one of the most prevalent gynecological malignancies, uterine corpus endometrial cancer (UCEC) brings about approximately 200000 diagnosed cases and 76000 female deaths all over the world annually 1, 2. With all the innovating diagnosis, drugs and therapies emerging, the incidence of UCEC is rising 3, and the 5-year survival rate of advanced UCEC patients is below 20%, which reflects a very poor prognosis 4. Hence, novel insights into the molecular mechanisms and genetic characteristics of UCEC progression are urgently demanded.

It has been a well-established consensus that overdosed estrogen exposure is a risk factor for UCEC, particularly the endometroid type 5, which represents 80% of total UCEC cases 6. Encoded by ESR1 and ESR2 gene respectively, estrogen receptors alpha (ERα) and estrogen receptor beta (ERβ) are classical intranuclear estrogen receptors in normal endometrium and UCEC cells 7, and regulate several cell processes, like cell growth, differentiation, etc. 8. To date, the functions of ERα is widely accepted as a tumor-promoter in hormone-responsive cancer, while the controversial role of ERβ is yet to be further elucidated.

MicroRNAs (miRNAs) refer to small non-coding RNAs modulating protein accumulation post-transcriptionally 9 through inhibition of messenger RNA (mRNA) translation or acceleration of mRNA degradation 10. MiR-765 has been reported to play a part in the progression of several cancers, such as osteosarcoma 11, clear cell renal cell carcinoma 12, gastric cancer 13, and breast cancer 14. However, no attention has been paid to the biofunctions of miR-765 in UCEC so far.

Cell-cell interaction in the tumor microenvironment has been shown to directly affect tumor progression and metastasis in many ways 15. Apart from cancer cells, numerous cell types play their indispensable roles in the tumor microenvironment, such as bone marrow-derived inflammatory cells, lymphocytes, blood vessels, fibroblastic cells, etc 16. It has been widely accepted that the infiltrates of CD8^+^ T cells into the cancer niche are linked to better prognosis for patients in a number of human malignancies including UCEC 17. As for CD8^+^ T cells, in addition to the classical tumor-killing mechanisms, emerging evidence has suggested that they exert effects on tumor by delivering exosomes to the recipient tumor cells, thus inducing the release of specific cargos, such as such mRNA, miRNA, proteins, and lipids 18. However, researches on CD8^+^ T cell-derived exosomal miRNAs in UCEC are still scarce.

The current study is to investigate the role and crosstalk of miR-765, estrogen and CD8^+^ T cell-derived exosomes of tumor microenvironment in the pathogenesis of UCEC *in vitro* and *in vivo*, and identify potential biomarkers and reveal novel molecular mechanisms for UCEC.

## Results

### Suppression of miR-765 level accelerates the proliferation and EMT of UCEC

**Figure 1:**
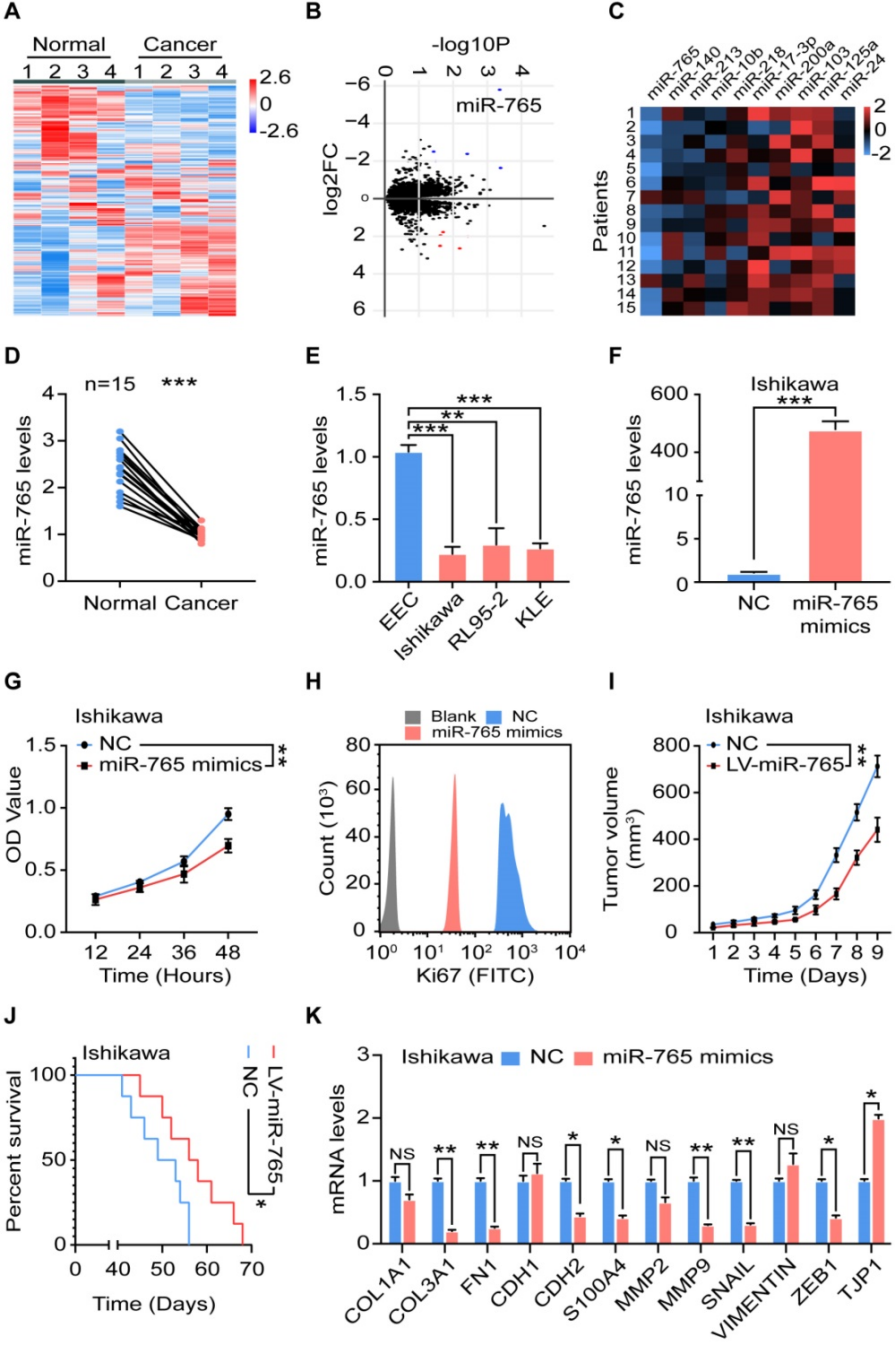
** Suppression of miR-765 level accelerates the proliferation and EMT of UCEC. A** Heatmap and **B** Volcano plot of miRNA-seq data for differentially expressed genes between normal (n = 4) and cancer (n = 4) tissues of UCEC. **C** Levels of top 5 up- and down-regulated miRNAs in clinic samples by RT-qPCR (n = 15). **D** Level of miR-765 in normal and cancer tissues by RT-qPCR. **E** Level of miR-765 in normal endometrial epithelial cells (EEC) and UCEC cell lines (Ishikawa, RL95-2, KLE cells) by RT-qPCR. **F** Level of miR-765 after treated with miR-765 mimics by RT-qPCR in Ishikawa. **G** The viability of Ishikawa cells was measured by the CCK-8 assay at 12 h, 24 h, 36 h, 48 h after treated with miR-765 mimics. **H** After treated with miR-765 mimics, the Ki67 expression of Ishikawa cells was determined by flow cytometry. **I** Tumor volume growth of NC and LV-miR-765 Ishikawa cells xenografts in mice were measured every day.** J** Survival curves of NC and LV-miR-765 Ishikawa cells xenografts-bearing mice. **K** mRNA levels of EMT related markers (*COL1A, COL3A1, FN1, CDH1, CDH2, S100A, MMP2, MMP9, SNAIL, VIMENTIN, ZEB1, TJP1*) in NC and miR-765 mimics-treated Ishikawa cells by RT-qPCR. Data were presented as mean ± SEM and analyzed by t test or ANOVA. * P < 0.05, ** P < 0.01, *** P < 0.001, NS: no significance.

To explore potential aberrant miRNAs in endometrial cancer, we analyzed gene microarray of cancer tissues (n = 4) and normal tissues (n = 4) (Figure 1A). Differential miRNAs were shown in the volcano plot (Figure 1B). Among these differentially expressed miRNAs, the top five up- and down-regulated (e.g., miR-765) miRNAs were verified by RT-qPCR (n = 15) (Figure 1B-C). Compared with normal paracancerous tissues, there was a significant decrease of miR-765 in UCEC clinical specimens (n = 15) (Figure 1D). Similarly, three kinds of UCEC cell lines (Ishikawa, RL95-2 and KLE cells) had the lower levels of miRNA-765 (Figure 1E).

To further evaluate the role of miR-765 in UCEC, miR-765-overexpressed Ishikawa and KLE cells were constructed by mimics transfection (Figure 1F and Figure S1A). As shown, miR-765 mimics led to reduced cell proliferation and Ki-67 expression in Ishikawa and KLE cells *in vitro* (Figure 1G-H and Figure S1B-C). *In vivo*, miR-765 mimics xenografts developed much slower and survived longer than those of NC group (Figure 1I-J, and Figure S1D-E). In addition, miR-765 mimics obviously down-regulated the expression of several EMT-related markers (e.g., *COL3A1, FN1, CDH2, S100A4, MMP9, SNAIL* and *ZEB1*) and up-regulated *TJP1* expression in tumor lesions (Figure 1K and Figure S1F). These data suggest that miR-765 should be a tumor suppressor gene for inhibiting cell proliferation and EMT process of UCEC.

### MiR-765 negatively regulates PLP2 expression of UCEC in a miRNAs cluster regulatory manner

**Figure 2:**
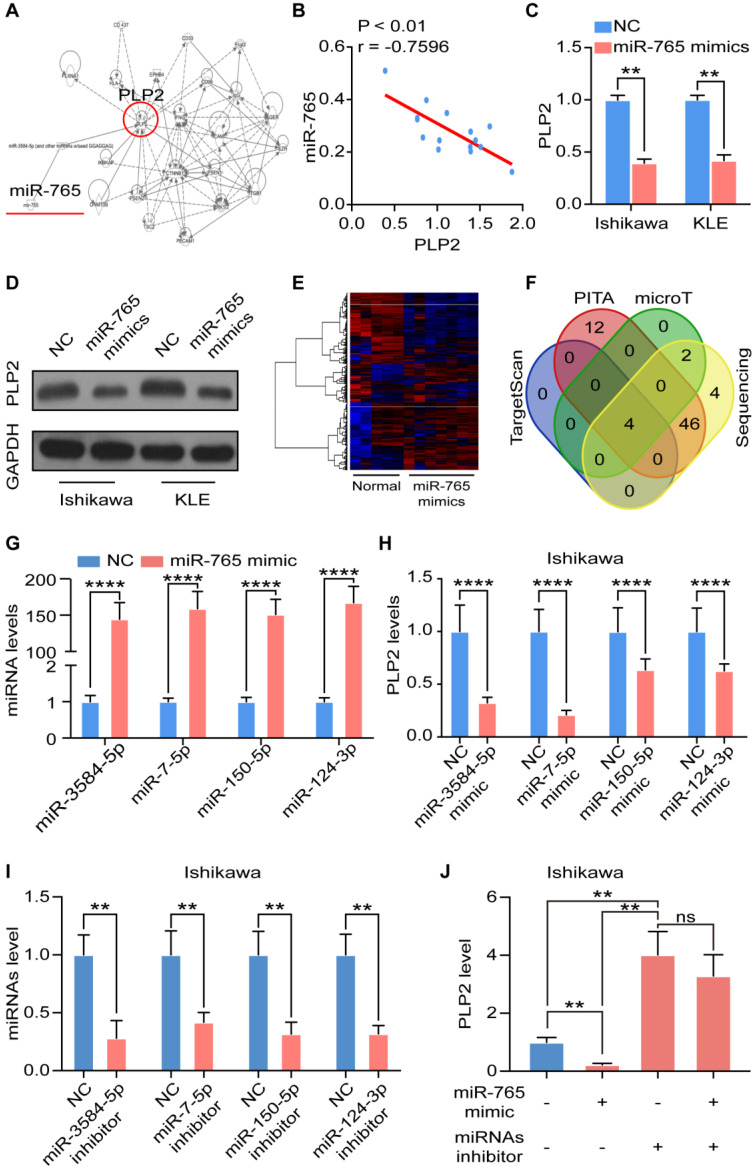
** MiR-765 negatively regulates PLP2 expression of UCEC in a miRNAs cluster regulatory manner. A** Bioinformatics analysis found that miR-765 inhibits PLP2 through miRNA clustering effect. **B** Spearman's correlation analysis for the correlation of miRNA-765 and PLP2 in UCEC tissues (n = 15) (R = -0.7596, p < 0.01). **C-D** RT-qPCR and western blotting were used to evaluate the mRNA and protein levels of PLP2 respectively after Ishikawa and KLE cells were treated with miR-765 mimics. **E** Heatmap of miRNA-seq data for differentially expressed genes between NC and miR-765 mimics-treated Ishikawa cells. **F** Venn diagram of intersection of miR-765 target genes predicted by bioinformatics analysis. **G** Levels of target miRNAs (miR-3584-5p, miR-7-5p, miR-150-5p and miR-124-3p) after treated with miR-765 mimics in Ishikawa cells by RT-qPCR. **H** Levels of PLP2 after treated with miR-3584-5p, miR-7-5p, miR-150-5p and miR-124-3p mimics in Ishikawa cells by RT-qPCR.** I** Levels of target miRNAs (miR-3584-5p, miR-7-5p, miR-150-5p and miR-124-3p) were assessed to evaluated the efficiency of miRNA inhibitors in Ishikawa cell by RT-qPCR. **J** Levels of PLP2 after treated with miR-765 mimics and/or miRNA inhibitors in Ishikawa cell by RT-qPCR. Data were presented as mean ± SEM and analyzed by t test or ANOVA. * P < 0.05, ** P < 0.01, *** P < 0.001, NS: no significance.

To search potential downstream targets of miR-765, we conducted bioinformatics analysis and the results showed that miR-765 might regulate Proteolipid protein 2 (PLP2) indirectly through miRNAs-cluster effects (Figure 2A). PLP2, a four-transmembrane domain protein located in the endoplasmic reticulum 19, 20, has been regarded as an oncogenic-inducer in several cancers including melanoma, osteosarcoma, breast cancer, hepatocellular carcinomas, and acute lymphoblastic leukemia 20-22. Notably, a negative correlation between the expression of miR-765 and PLP2 was corroborated in 15 cancer tissues (r = -0.7596, P < 0.01) (Figure 2B). RT-qPCR assays showed that miR-765 mimics inhibited PLP2 transcription in Ishikawa and KLE cells (Figure 2C). In addition, data of western blotting showed that miR-765 mimics markedly decreased the expression of PLP2 in Ishikawa and KLE cells (Figure 2D and Figure S2A). These results indicate that miR-765 negatively regulates PLP2 expression.

Subsequently, miRNA sequencing was performed on NC and miR-765 mimics treated Ishikawa cells to screen the potential intermediate regulators between miR-765 and PLP2 (Figure 2E). By means of a combined analysis of TargetScan (http://www.targetscan.org/), PITA (http://genie.weizmann.ac.il/pubs/mir07/mir07_data.html), microT (http://www.microrna.gr/microT) databases and miRNA sequencing data, 4 miRNAs directly targeting PLP2 were screened out, including miR-3584-5p, miR-7-5p, miR-150-5p and miR-124-3p (Figure 2F). We explored the specific mechanism and found that miR-765 regulated several transcription factors, which were closely related with transcription of miR-3584-5p, miR-7-5p, miR-150-5p and miR-124-3p (Figure S2B). Indeed, miR-765 mimics significantly enhanced the levels of these miRNAs in both Ishikawa and KLE cells (Figure 2G and Figure S2C). With the transfection of mimics of these miRNAs in Ishikawa and KLE cell lines, the level of PLP2 decreased consistently (Figure 2H and Figure S2D). Furthermore, we tested the effects of miRNA inhibitors on Ishikawa and KLE cells and found that miRNA inhibitors could effectively inhibit miRNA levels (Figure 2I and Figure S2E). Interestingly, the inhibitory effect of miR-765 mimics on the level of PLP2 in Ishikawa and KLE cell lines could be completely abrogated by miRNA inhibitors (Figure 2J and Figure S2F). These data suggest that the negative regulation of miR-765 on PLP2 should be dependent on the miRNAs cluster (e.g., miR-3584-5p, miR-7-5p, miR-150-5p and miR-124-3p) in UCEC cells.

### PLP2 promotes the EMT process and invasion of UCEC by activation of Notch signaling

**Figure 3:**
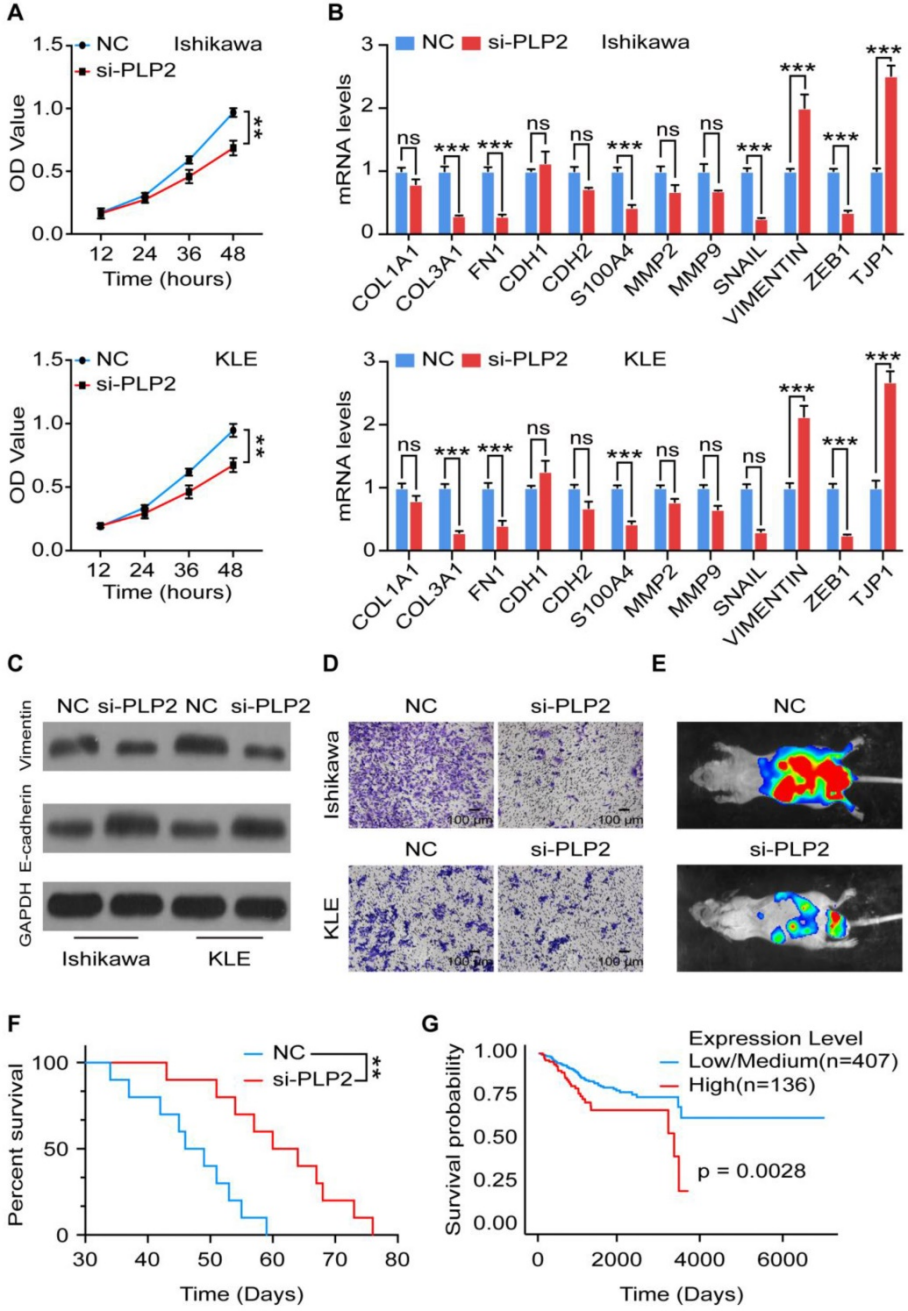
** PLP2 promotes the EMT process and invasion of UCEC. A** Cell viability of NC or siPLP2 Ishikawa (up) and KLE (down) cells was measured by the CCK-8 assay at 12 h, 24 h, 36 h, 48 h. **B** mRNA levels of EMT related markers (*COL1A, COL3A1, FN1, CDH1, CDH2, S100A, MMP2, MMP9, SNAIL, VIMENTIN, ZEB1, TJP1*) in NC or siPLP2 Ishikawa and KLE cells by RT-qPCR. **C** Western blotting images of EMT-related markers (E-cadherin and Vimentin) in NC or siPLP2 Ishikawa and KLE cells. **D** Cell invasion was detected in NC or siPLP Ishikawa and KLE cells at 24 h. Scale bars: 100 µm. **E** *In vivo* imaging of tumor metastasis after injection of NC or siPLP2 transfected Ishikawa cells in mice.** F** Survival curves of NC and siPLP2 Ishikawa cells xenografts-bearing mice. **G** Kaplan-Meier plots were drawn to visualize the survival outcomes for UCEC patient with high or low level of PLP2 (p = 0.0028).

To explore the role of PLP2 in UCEC, we knocked down the expression of PLP2 and verified the efficiency by RT-qPCR and western blotting (Figure S3A-C). As shown, silencing PLP2 significantly suppressed the proliferation of Ishikawa and KLE cells (Figure 3A), and induced an impaired EMT process, which was not only confirmed by downregulation of *COL3, FN1, S100A4, SNAIL, ZEB1* as well as upregulation of *TJP1* at the mRNA level (Figure 3B), but also by downregulation of Vimentin and upregulation of E-cadherin at the protein level in Ishikawa and KLE cells (Figure 3C and Figure S3D-E). Additionally, absence of PLP2 inhibited the invasion of Ishikawa and KLE cells (Figure 3D).* In vivo,* we also observed that PLP2 knockdown suppressed the metastasis of Ishikawa cells which were intravenously injected into mice and prolonged the survival of tumor-bearing mice (Figure 3E-F). According to the TCGA database, in particular, low level of PLP2 was associated with a better prognosis in UCEC patients (Figure 3G). Taken together, these results indicate that PLP2 should be a tumor-promoting protein by accelerating cell proliferation, EMT process, invasion and metastasis in UCEC.

**Figure 4:**
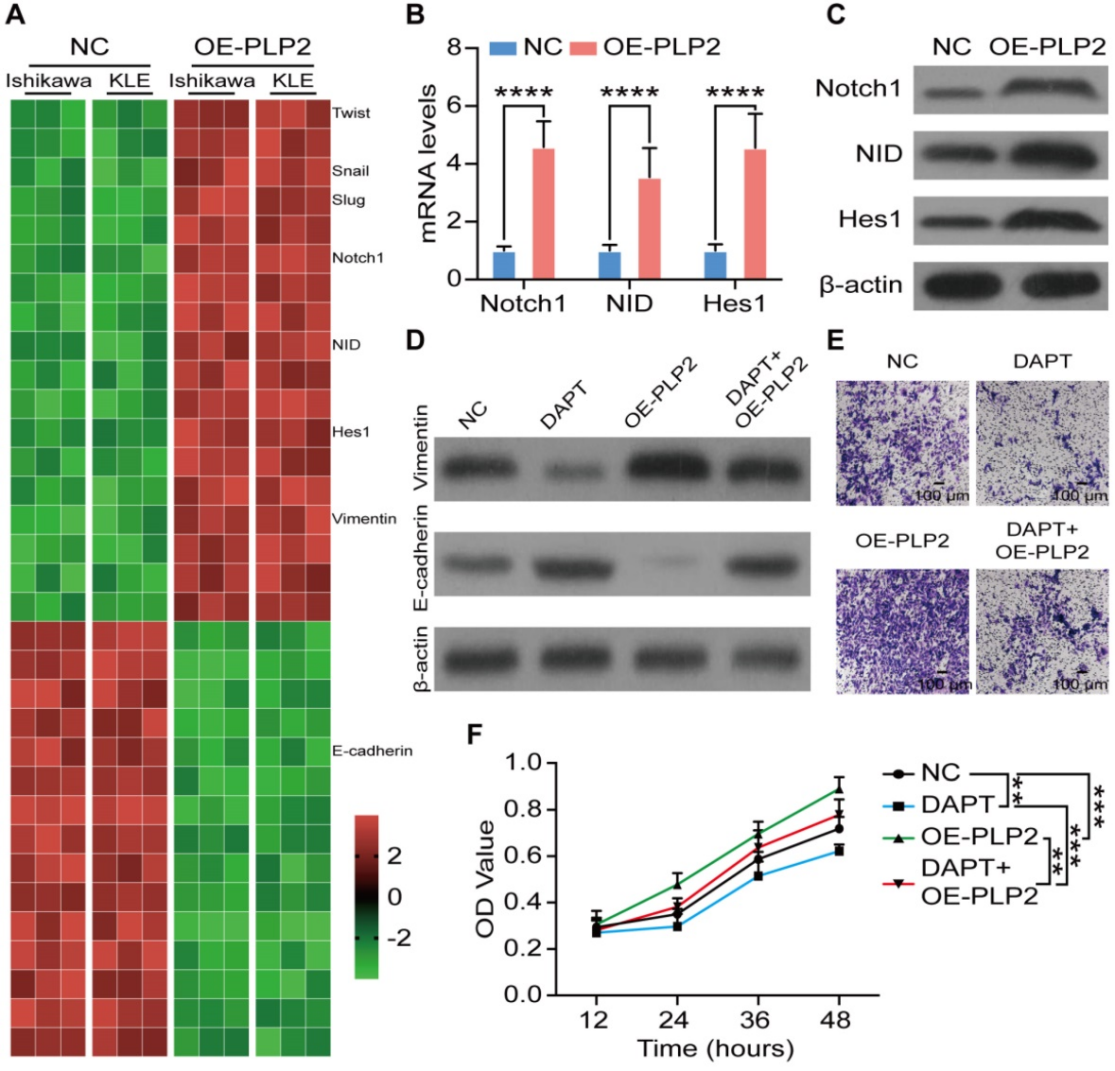
** PLP2 enhances the EMT process of UCEC by activation of Notch signaling. A** Heatmap of mRNA-seq data for differentially expressed genes between NC and OE-PLP2 Ishikawa and KLE cells.** B-C** mRNA and protein levels of Notch-related molecules (Notch1, NID, Hes1) in NC and OE-PLP2 Ishikawa cells by RT-qPCR. After NC or OE-PLP2 Ishikawa cells after treated with DAPT or not, levels of EMT related markers (E-cadherin and Vimentin) **(D),** cell invasion **(E)** and cell viability **(F)** were detected. Data were presented as mean ± SEM and analyzed by t test or ANOVA. * P < 0.05, ** P < 0.01, *** P < 0.001, NS: no significance. Scale bars: 100 µm.

To further uncover the potential mechanism of PLP2 on EMT in UCEC cells, RNA sequencing was performed in NC and PLP2-overexpressed (OE-PLP2) Ishikawa and KLE cells. As depicted in Figure 4A, the enrichment analysis of differential expression genes showed that EMT-related genes, *NOTCH1*, *NID* and *HES1* were significantly up-regulated in OE-PLP2 group. Next, we observed that PLP2 obviously promoted the activation of Notch signaling in Ishikawa and KLE cells (Figure 4B-C and Figure S4A-B). Additionally, DAPT, an inhibitor for Notch signaling pathway, could significantly restrict the stimulatory effect of OE-PLP2 on EMT-related molecules, cell invasion and proliferation* in vitro* (Figure 4D-F and Figure S4C-E). These data suggest that PLP2-meditaed EMT process and invasion of UCEC cells are dependent on the activation of Notch signaling pathway.

### Estrogen regulates the miR-765/PLP2 level in UCEC through the ERβ

**Figure 5:**
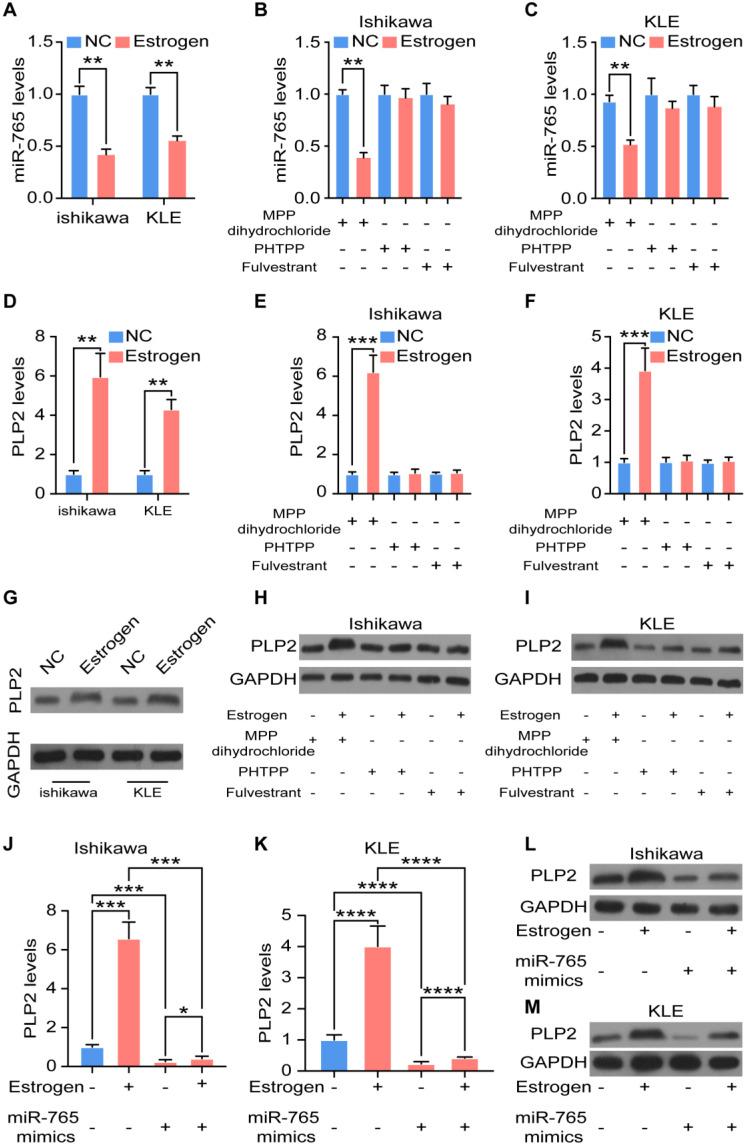
** Estrogen regulates the miR-765/PLP2 level in UCEC through the ERβ. A** Levels of miR-765 after Ishikawa and KLE cells were treated with estrogen by RT-qPCR. **B-C** Levels of miR-765 were assessed after Ishikawa and KLE cells were treated with estrogen and MPP or PHTPP or Fulvestrant by RT-qPCR. **D** Levels of PLP2 were tested after Ishikawa and KLE cells were treated with estrogen by RT-qPCR. **E-F** Levels of PLP2 were assessed after Ishikawa and KLE cells were treated with estrogen and MPP or PHTPP or Fulvestrant by RT-qPCR. The relevant protein levels of PLP2 were measured by western blotting in **(G-I). J-M** RT-qPCR and western blotting were conducted to evaluate the levels of PLP2 in Ishikawa and KLE cells after treated with estrogen and/or miR-765 mimics. Data were presented as mean ± SEM and analyzed by t test or ANOVA. * P < 0.05, ** P < 0.01, *** P < 0.001, **** P < 0.0001, NS: no significance.

In term of the vital driving factors of endometrial cancer, the effect of estrogen on miR-765/PLP2 levels was evaluated. As expected, stimulation with estrogen significantly decreased the expression of miR-765 in Ishikawa and KLE cells (Figure 5A). However, these effects could be reversed by PHTPP (an ERβ inhibitor), and Fulvestrant (an ER inhibitor), but not MPP (an ERα inhibitor) (Figure 5B-C). In contrast, estrogen up-regulated PLP2 expression, and the application of PHTPP and Fulvestrant rather than MPP could block this effect (Figure 5D-I and Figure S5A-C). Meanwhile, the up-regulation of PLP2 induced by estrogen could partly reversed by miR-765 mimics in Ishikawa and KLE cells (Figure 5J-M and Figure S5D-E).

In treatment of advanced breast cancer, the selective estrogen receptor degrader fulvestrant, has been confirmed by improved efficacy and decreased side effects 23. As shown, fulvestrant significantly down-regulated the expression of both ESR1 (coding gene of ERα) and ESR2 (coding gene of ERβ) in Ishikawa (ERα^+^, ERβ^+^) and KLE (ERα^-^, ERβ^+^) cells (Figure S5F-G). Subsequently, we further evaluated the possible treatment value of fulvestrant in UCEC, and found that fulvestrant alone up-regulated the expression of miR-765 (Figure S5H), but down-regulated the level of PLP2 (Figure S5I-K) in Ishikawa and KLE cells. Additionally, the knockdown of ERβ (si-ERβ) could partly abolished the regulatory effects of estrogen on miR-765 and PLP2 in Ishikawa and KLE cells (Figure S5L-M). These data demonstrate that estrogen regulates the miR-765 and PLP2 levels, and fulvestrant can restrict estrogen-driven regulation of miR-765 and PLP2 levels in UCEC cells in ERβ-dependent and -independent manners.

### CD45RO^-^CD8^+^ T cell-derived exosomal miR-765 partly suppressed PLP2 in UCEC cells

**Figure 6:**
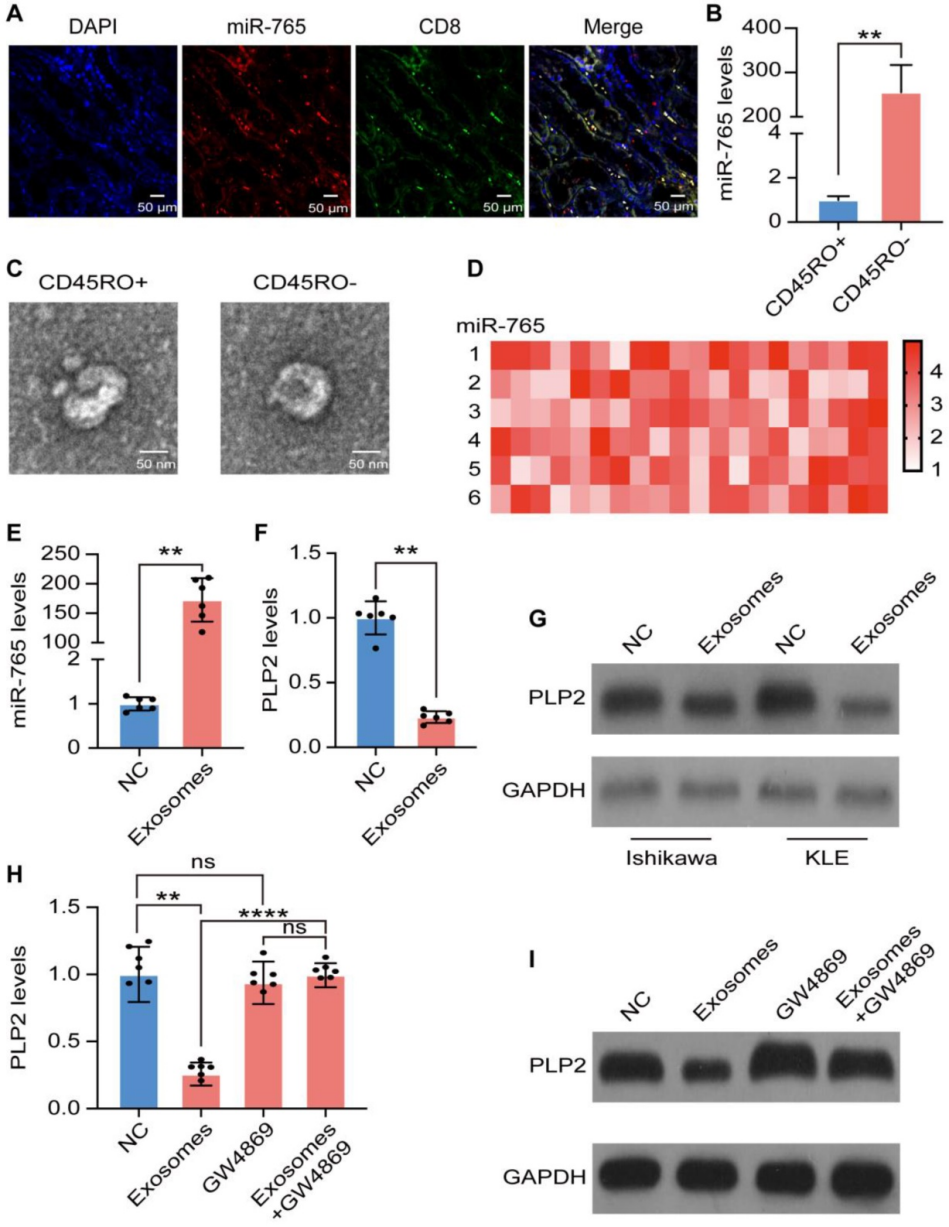
** CD45RO^-^CD8^+^ T cell-derived exosomal miR-765 partly suppresses PLP2 in UCEC cells. A** Immunofluorescence of nucleus (blue), CD8 (green) and miR-765 (red) were shown in UCEC tissues. A co-localization between CD8 and miR-765 was depicted as yellow in merged images. Scale bars: 50 µm. **B** Levels of miR-765 were tested in CD45RO^+^CD8^+^ and CD45RO^-^CD8^+^ T cells by RT-qPCR. **C** Identification of exosomes from CD45RO^+^CD8^+^ T cells and CD45RO^-^CD8^+^ T cells by TEM. Scale bars: 50 nm. **D** Heatmap of CD45RO^-^CD8^+^ T cells exosomal miRNAs levels by miRNA sequencing. **E** Levels of miR-765 in Ishikawa were tested after treated with CD45RO^-^CD8^+^ T cell-derived exosomes by RT-qPCR. **F-G** mRNA and protein levels of PLP2 in Ishikawa were tested after treated with CD45RO^-^CD8^+^ T cell-derived exosomes by RT-qPCR and western blotting. **H-I** mRNA and protein levels of PLP2 in Ishikawa were tested after treated with CD45RO^-^CD8^+^ T cell-derived exosomes and/or GW4869 by RT-qPCR and western blotting. Data were presented as mean ± SEM and analyzed by t test or ANOVA. * P < 0.05, ** P < 0.01, *** P < 0.001, **** P < 0.0001, NS: no significance.

Since CD8^+^ T cells function essentially in anti-tumor immunity, combined fluorescent *in situ* hybridization for miR-765 and immunofluorescent labeling for CD8 were carried out and the evidence of co-localization between miR-765 and CD8^+^ T cell was presented (Figure 6A), revealing that miR-765 should be highly expressed in CD8^+^ T cells of UCEC lesions. To determine the possible source of miR-765 in CD8^+^ T cells, CD45RO^+^CD8^+^ T cells and CD45RO^-^CD8^+^ T cells were sorted by FACS (Figure S6A). Of note, RT-qPCR assay showed that a much higher level of miR-765 in CD45RO^-^CD8^+^ T cells was observed comparing with CD45RO^+^CD8^+^ T cells (Figure 6B). To explore the source of miR-765, exosomes of CD45RO^-^CD8^+^ T cells and CD45RO^+^CD8^+^ T cells were purified and identified by TEM, flow cytometry and western blotting (abundant expression of CD63 and TSG101, and the absence of GRP78) respectively (Figure 6C and Figure S6B-F). Meanwhile, miRNA sequencing data of exosomal contents identified miR-765 as the most abundant miRNA in CD45RO^-^CD8^+^ T cell-derived exosomes (Figure 6D). Interestingly, exosomes from CD45RO^-^CD8^+^ T cells significantly up-regulated miR-765 and down-regulated PLP2 levels in Ishikawa and KLE cells (Figure 6E-G and Figure S6G-I). However, the negative regulatory effect of exosomes on PLP2 levels in Ishikawa and KLE cells was completely abolished by GW4869 (an exosomes inhibitor) (Figure 6H-I and Figure S6J-K). Briefly, these results demonstrate that CD45RO^-^CD8^+^ T cell-derived exosomal miR-765 can suppress PLP2 expression in UCEC.

### Exosomes originated from CD45RO^-^CD8^+^ T cell partly suppress UCEC development in a miR-765/PLP2-dependent manner

**Figure 7:**
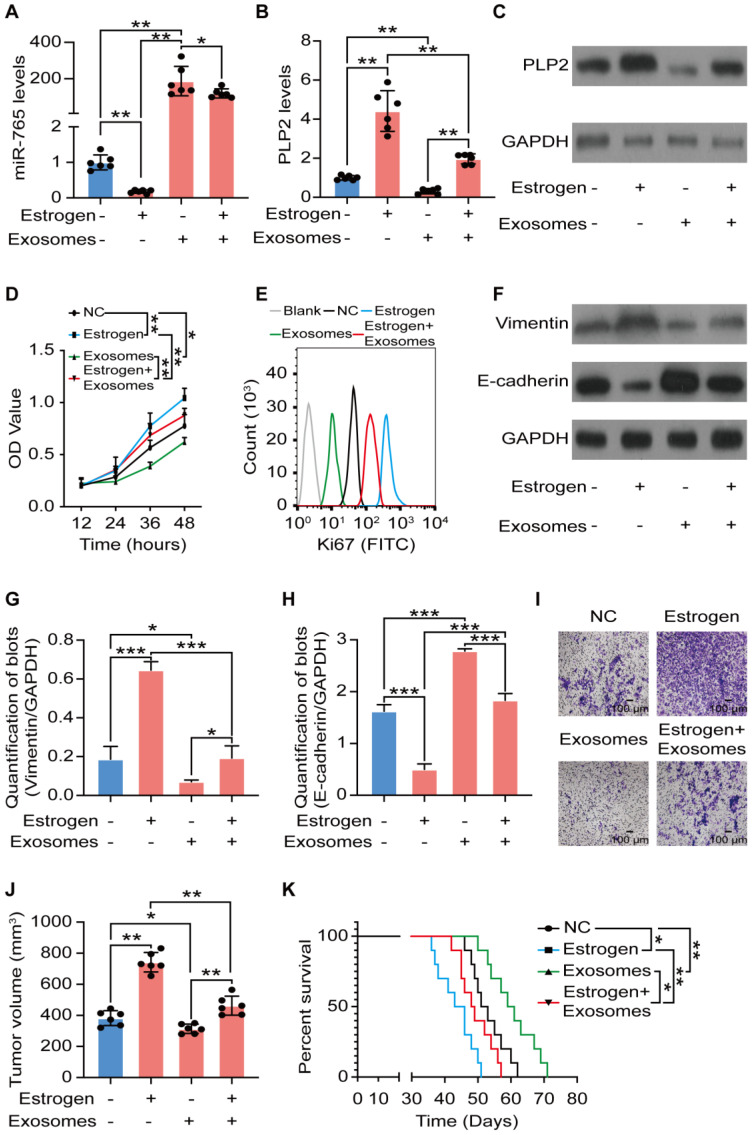
** Exosomes originated from CD45RO^-^CD8^+^ T cells partly suppress UCEC development in a miR-765/PLP2-dependent manner.** After Ishikawa cells were treated with estrogen and/or CD45RO^-^CD8^+^T cell-derived exosomes for 24 h, **A** Levels of miR-765 were tested by RT-qPCR. **B-C** Levels of PLP2 were tested by RT-qPCR and western blotting. **D** Cell viability was measured by the CCK-8 assay at 12 h, 24 h, 36 h or 48 h. **E** Ki67 expression was determined with by flow cytometry. **F** Levels of EMT related markers (Vimentin and E-cadherin) were measured by western blotting and were quantified in** (G-H)**, respectively. **I** Cell invasion was detected by the transwell assays. Scale bars: 100 µm. **J** Ultimate tumor volume of Ishikawa cells xenografts in mice were measured. **K** Survival curves of Ishikawa cells xenografts-bearing mice was plotted. Data were presented as mean ± SEM and analyzed by t test or ANOVA. * P < 0.05, ** P < 0.01, *** P < 0.001, **** P < 0.0001, NS: no significance.

Owing to the important roles of hormone-immune microenvironment on UCEC development, the dialogue between exosomes of CD45RO^-^CD8^+^ T cells and estrogen signaling in UCEC was evaluated. As illustrated in Figure 7A-C, and Figure S7A-C, exosomes of CD45RO^-^CD8^+^ T cells alone significantly up-regulated miR-765 but down-regulated PLP2 levels in Ishikawa and KLE cells. Additionally, exosomes partly or completely reversed estrogen-induced regulatory effects on miR-765 and PLP2 levels in Ishikawa and KLE cells. Subsequently, we observed that exosomes partly or completely abolished the stimulatory effects of estrogen on cell proliferation, Ki67 expression, EMT process and invasion of Ishikawa and KLE cells *in vitro* (Figure 7D-I and Figure S7D-I). Most noteworthy, treatment with exosomes alleviated the estrogen-induced tumor growth and poor prognosis of Ishikawa and KLE cells xenografts bearing mice (Figure 7J-K and Figure S7J-K). These data illustrate that CD45RO^-^CD8^+^ T-derived exosomes partially restricts estrogen-driven UCEC development via regulation of the miR-765/PLP2 axis.

## Discussion

As an indispensable hormone for both gender, estrogen plays pivotal roles in many physiological processes, including metabolism, immune responses, bone or cardiovascular health, reproductive functions, etc. 24. However, abnormal levels of estrogen in human body, which derived from either endogenous production or exogenous ingestion, may lead to benign diseases or even malignant cancers. For instance, high level of estrogen may lead to obesity 25, endometriosis 26, UCEC, breast cancer 27, etc. While osteoporosis 27 and diabetes 28 should be attributed to the decrease of estrogen. Generally, estrogen plays its roles through estrogen receptors (ER). As the ligand-bound ERα promotes growth of hormone-responsive cancer, the ERβ levels and/or the ERβ/ERα ratio decreases during the tumorigenesis of some malignant cancers 29. Moreover, ERβ has been reported to inhibit the proliferation of prostate and breast cancer, which indicates the tumor-suppressor role of ERβ 30, 31, Accumulated studies have uncovered the critical role of estrogen in the tumorigenesis and development of UCEC 32. However, the role and mechanism of ERβ signaling in UCEC remain largely unknown. In our study, we observed estrogen reduced miR-765 levels and increased PLP2 levels, further contributing to the EMT process and metastasis of UCEC. Interestingly, PHTPP and Fulvestrant could partly reverse these effects, suggesting estrogen-mediated miR-765/PLP2 expression regulation and UCEC development should be achieved in ERβ-dependent and independent manners. Apart from the classic ERα and ERβ, several non-classic estrogen receptors and their variants have been unveiled to the public, such as G-protein-coupled estrogen receptor-1 (GPER-1) 33, estrogen receptor-related receptors (ERRs) 34, and ERα-36 35. However, other mechanisms of Fulvestrant on blocking the estrogen-miR-765/PLP2 axis need to be further clarified.

Despite that UCEC has been already categorized as an estrogen-motivated disease, it is also recognized as a genetic disease 36. It has been reported that miR-765 has dual regulatory functions on several cancers. Here, we found that there was a negative correlation between miR-765 and PLP2 in UCEC patients. Further analysis showed that absence of miR-765 led to higher proliferation, EMT process, invasion and development of UCEC by upregulation of PLP2. Different from a previous report 12, the negative regulatory effect of miR-765 on PLP2 expression was indirect in UCEC cells. Notably, other miRNAs (e.g., miR-150-5p and miR-124-3p) also involved in targeting PLP2 37, 38. Owing to the bioinformatics analysis and further analysis, it can be speculated that intermediate regulators should be involved in this process, including miR-3584-5p, miR-7-5p, miR-150-5p and miR-124-3p. However, the detailed molecular mechanism remains to be further explored.

EMT, the process in which epithelial cells obtain mesenchymal features, is associated with tumor initiation, invasion, metastasis, and resistance to therapy in cancer 39. Currently, the relationship between miRNAs and EMT is widely acknowledged in a variety of cancers. However, the role of miRNAs in the EMT of UCEC is poorly understood 40. The only reports suggest that both miR-194 and miR-200c suppress BMI-1 directly, thus resulting in the inhibition of PTEN or transcription factors ZEB1 and ZEB2 to preserve the epithelial phenotype in UCEC 41, 42. As for the involvement of miR-765 in EMT, Lv *et al* reported that via activating the ERK, miR-765 promoted the progression and stimulate EMT process of osteosarcoma 43. Notably, our data showed that both the upregulation of miR-765 and the succedent downregulation of PLP2 resulted in the suppression of EMT in UCEC, which was reflected by the downregulation of Vimentin and the upregulation of E-cadherin. Additionally, under the positive regulation of estrogen, PLP2 accelerates the invasion and metastasis of UCEC *in vitro* and *in vivo.* The Notch signaling pathway has been reported to participates in tumorigenesis, including regulation of EMT 44. In current studies, the data of RNA sequencing and further analysis indicates that the stimulatory effect of PLP2 on EMT, invasion and development of UCEC should be dependent on the activation of Notch signaling pathway. Although the molecule mechanism of PLP2 on Notch signaling pathway still unclear, the evidence above provides novel insights into the associations among estrogen, miR-765/PLP2, Notch signaling and EMT in UCEC progression.

One of the pivotal steps in the process of host anti-tumor immunity is the activation of CD8^+^T cells, during which numerous membrane vesicular bodies termed exosomes are released 45. It has been reported that T cells release exosomes containing miR-142-3p, miR-142-5p, and miR-155, which can be transferred in active form to β cells favoring apoptosis, thus contributes to Type 1 Diabetes Development 46. Naohiro *et al* reported that cytotoxic miR-298-5p-embedded exosomes released by CD8^+^ T cells from normal mice could kill lesional mesenchymal cells in fibroblastic tumor 47. A high level of infiltrating and circulating CD45RO^+^CD8^+^ T cells in metastases has been found to be favorable for the outcome of different tumors 48, 49. In current study, we observed CD8^+^ T cells and miR-765 were co-located in UCEC lesions. It is worthy to point out that CD45RO^-^ naïve CD8^+^ T cells possessed higher level of miR-765 compared with CD45RO^+^ memory CD8^+^ T cell subsets. Exosomes released by CD45RO^-^CD8^+^ T cells could up-regulated the levels of miR-765 in UCEC, and further decrease PLP2 expression, contributing to impaired cell proliferation, invasion, EMT process, and better outcomes of UCEC *in vivo*. In addition, these exosomes could partly or completely restrict estrogen-triggered UCEC development by regulating the miR-765/PLP2 axis, indicating that there should be a potential value of CD45RO^-^CD8^+^ T cell-released exosomes for treatment of UCEC. However, it is unclear whether there are other molecules from CD45RO^-^CD8^+^ T cell-released exosomes that can cooperate with miR-765 in the regulation of anti-estrogen effects in UCEC. Additionally, the regulatory factors for exosomes release from CD8^+^ T cell in local UCEC lesions need to be further explored.

## Conclusion

**Figure 8:**
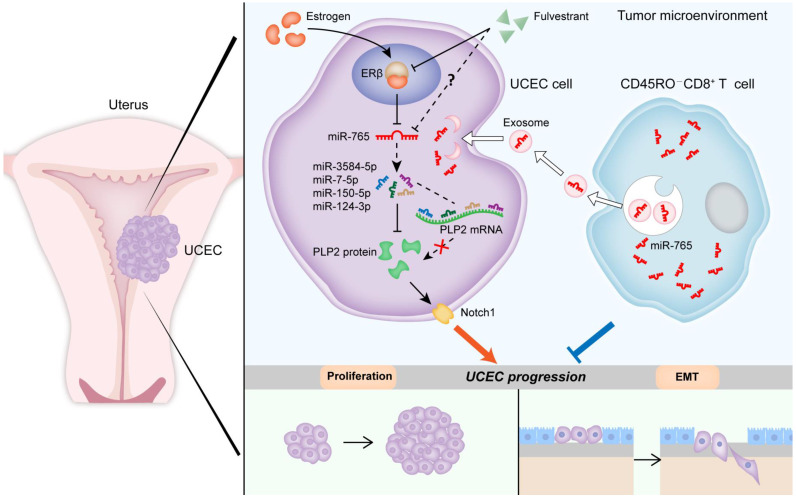
** Schematic illustration of the mechanisms by which CD45RO^-^CD8^+^ T cell-derived exosomes restrict estrogen-driven endometrial cancer development via the ERβ/miR-765/PLP2/Notch axis.** In UCEC cells, estrogen significantly decreases miR-765 level, and facilitates the development of UCEC by ERβ through the miRNAs (e.g., miR-3584-5p, miR-7-5p, miR-150-5p and miR-124-3p) cluster-controlled regulation of the PLP2, thus promoting the proliferation and EMT process in a Notch signaling pathway-dependent manner. Interestingly, the selective ER degrader Fulvestrant alleviates estrogen-mediated miR-765/PLP2 expression regulation and UCEC development in ERβ-dependent and independent manners. CD45RO^-^CD8^+^ T cell-derived exosomes restrict estrogen-driven disease development via regulation of the miR-765/PLP2 axis. The solid arrows represent direct processes; the dashed arrows represent indirect processes.

Collectively, as shown in Figure 8, our data *in vitro* and* in vivo* suggest that aberrant low level of miR-765 leads to high proliferation, EMT process, invasion and poor prognosis of UCEC by the activation of PLP2-Notch signaling pathway. As a typical risk factor of UCEC, estrogen/ERβ regulates the miR-765/PLP2 axis and further accelerates disease development. Most importantly, Fulvestrant can reverse estrogen-mediated miR-765/PLP2 expression regulation and UCEC development in ERβ-dependent and independent manners, further explaining the new mechanism of Fulvestrant on UCEC treatment. Additionally, CD45RO^-^CD8^+^ T cell-derived exosomes release high level of miR-765, and limit the tumor-promoting effects of estrogen on UCEC via regulation of the miR-765/PLP2 axis. Therefore, these findings provide evidence that the ERβ/miR-765/PLP2/Notch signaling axis regulates the progression of UCEC, and indicate a novel perspective on the anti-tumor mechanisms of CD8^+^ T cells and exosomes. In addition, the potential therapeutic value of CD45RO^-^CD8^+^ T cell-derived exosomes in UCEC should be emphasized due to the negative regulation of estrogen/miR-765/PLP2 axis-mediated disease development.

## Materials and methods

### Clinic samples

Signed informed consents were obtained from all the enrolled participants. Cancer tissues and adjacent normal tissues were collected from 15 endometrial cancer patients (35‐55 years old) whose surgical pathology specimens were identified as endometrial adenocarcinomas at the Department of Gynecology from May 2018 to August 2019. All tissues were preserved at -80 ℃ until use. This research was approved by the Ethics Committee of Obstetrics and Gynecology Hospital of Fudan University.

### Cell culture

Primary endometrial epithelial cells (EECs) were isolated from benign endometrium which were rinsed with phosphate buffered saline (PBS), minced into small pieces, and incubated in collagenase Ⅳ and DNase I in water bath at 37 °C. The cell suspensions were then filtered through cell strainers to separate epithelial cells. Ishikawa, RL95-2, and KLE cell lines were obtained from the Cell Bank of the Chinese Academy of Sciences (Shanghai, China). All the cells were cultured in DMEM/F12 (Gibco, Auckland, NZ) supplemented with 1% penicillin-streptomycin (HyClone, Utah, USA) and 10% certified FBS-charcoal-stripped (Biological Industries, Israel) at 37 °C with 5% CO_2_.

### Cell treatment and transfection

Ishikawa or KLE cells were treated with or without estrogen (10 nM; Sigma-Aldrich, 50-28-2), an ERα inhibitor (MPP dihydrochloride hydrate, 10 μM; Sigma-Aldrich, 911295-24-4), an ERβ inhibitor (PHTPP, 10 μM; Sigma-Aldrich, 805239-56-9) or an ER inhibitor for both ERα and ERβ (Fulvestrant, 250 nM; Sigma-Aldrich, 129453-61-8), an inhibitor for Notch signaling (DAPT, 50 μM; Sigma-Aldrich, 208255-80-5), or an exosomes inhibitor (GW4869, 10 μM; Sigma-Aldrich, 6823-69-4) for 48 h, and then the transcriptional levels and protein levels of specific molecules or the bio-functions were detected.

The GFP-labeled lentivirus vectors containing the has-miR-765 mimic lentivirus (miR-765 mimics) and the corresponding control miRNA lentivirus (miR-NC, negative control), the PLP2 over-expression lentivirus (OE-PLP2) and the corresponding control lentivirus (NC-PLP2) and the small interfering RNA si-PLP2 and the corresponding control si-NC were obtained from GeneChem (Shanghai, China). Transduction with the lentiviral vectors was conducted using transduction reagents and 8 mg/mL Polybrene (GeneChem) for 12 h. The siRNA transduction was conducted with Lipofectamine3000 Reagent according to the manufacturer's instructions (Invitrogen, Carlsbad, California, USA). After transfected into Ishikawa, RL95-2, and KLE cell lines, stable cell lines were then established, and the efficiency was confirmed by RT-qPCR.

### Cell viability assay

Cell Counting Kit‑8 (CCK‑8) reagent (Dojindo, Tokyo, Japan) was used to test cell viability according to the manufacturer's instructions. All the experiments were conducted at least in triplicate.

### Transwell assay

All the transwell chambers (Corning Incorporated, Corning, NY, USA) were coated with 50 µL Matrigel (354480; BD Biosciences) and incubated at 37 °C for 1 h in advance. Next, 5 × 10^4^ cells re-suspended in 200 µL FBS-free culture medium were seeded in the upper chamber. The bottom chamber was filled with 500 µL culture medium containing 20% FBS. After 24 h of incubation, a cotton swab was used to eliminate noninvasive cells in the upper chamber. Invasive cells were fixed in 4% paraformaldehyde, stained with 0.5% crystal violet, and imaged under an inverted microscope. Finally, invasive cells in five randomly selected fields were counted.

### Preparation and purification of CD8^+^ T cell-released exosomes

A total of 2 × 10^6^ cells/ml lymphocytes derived from peripheral blood of healthy human donors were cultured in RPMI 1640 complete medium (containing 50 mM 2-ME, 1 mM sodium pyruvate, 10 mM HEPES, and 10 U/mL IL-2). CD45RO^+^CD8^+^ or CD45RO^-^CD8^+^ T cells were purified by fluorescence activated cell sorting (FACS). Purified CD8^+^ T cells were then cultured in exosome-free RPMI 1640 complete medium for 48 h. Next, supernatants of cell culture medium were collected and then centrifuged at 800 × *g* for 5 min and followed by centrifugation of 2000 × *g* for 10 min to remove cellular fragments. Then, the medium was filtered through a 0.2 µm pore strainer (Syringe filter), and ultracentrifuged at 100000 × *g* for 2 h at 4 °C and then pelleted exosomes from CD45RO^+^CD8^+^ or CD45RO^-^CD8^+^ T cells were obtained.

### Transmission electron microscopy (TEM)

For transmission electron microscopy observations, exosome pellets were fixed in 4% paraformaldehyde at 4 °C for 1 h. Then, the pellets were loaded onto electron microscopy grids coated with formvar carbon, contrasted, and embedded in a mixture of uranyl acetate and methylcellulose. Sections were observed with a Philips Tecnai-10 transmission electron microscope operating at 80 kV (Phillips Electronic Instruments, Mahwah, NJ).

### Flow cytometry assays

Antibodies for flow cytometry assays were used for measurement of cell markers, which include FITC anti-mouse/human Ki-67 Antibody (151211, Biolegend, USA), APC anti-human CD8 antibody (980904, Biolegend, USA), PE anti-human CD28 antibody (302907, Biolegend, USA) and PE/Cyanine7 anti-human CD45RO Antibody (304229, Biolegend, USA). Matched immunoglobulin G (IgG) antibodies were used as isotype controls. Flow cytometry assays were performed using according to the manufacturer's instructions. Cell sorting was conducted with a Beckman CytoFLEX S flow cytometer (Beckman) using Becton CytExpert software. Data were analyzed using FlowJo V10 software.

### Reverse transcription-quantitative PCR (RT-qPCR)

Total RNA was extracted by TRIzol (Invitrogen, Carlsbad, California, USA) from tissues and cells, and the concentration was quantified by a NanoDrop spectrophotometer (NanoDrop Technologies; Thermo Fisher Scientific, Inc.). The PrimeScript™ RT Reagent Kit (TaKaRa Biotechnology, Co., Ltd., Dalian, China) was used to reversely transcribe total RNA to cDNA. Next, SYBR Green PCR Master Mix (TaKaRa Biotechnology) was used to perform RT-qPCR. The miScript Reverse Transcription Kit (Qiagen GmbH, Hilden, Germany) and miScript SYBR Green PCR Kit (Qiagen GmbH) were used to measure miR-765 level for reverse transcription and RT-qPCR, respectively. U6 acted as the endogenous control. All reactions were processed on the Applied Biosystems 7500 Real-Time PCR System (Thermo Fisher Scientific, Inc.). Relative gene expression was analyzed using the 2^-ΔΔCt^ method. The primers were listed in Table S1.

### miRNA-seq and mRNA-seq

Gene expression analysis was conducted by miRNA-seq and mRNA-seq for the conditions described in the relevant figures. For miRNA-seq, total miRNA was isolated using the miRNeasy Mini Kit (Qiagen GmbH, Hilden, Germany) according to the manufacturer's instructions. Quality of the total RNA was measured by the Agilent 2100 Bioanalyzer and samples with a RNA integrity number (RIN) higher than 7 were used for sequencing. cDNA libraries were generated using the NEBNext Multiplex Small RNA Library Prep Set for Illumina (New England Biolabs). Libraries were size selected using a 6% polyacrylamide gel and purified using the QIAQuick PCR Purification Kit (Qiagen GmbH). Purified libraries were normalized and pooled to create a double stranded cDNA library ready for sequencing. The samples were sequenced on the Illumina MiSeq to render 50 base pair single end reads. For mRNA-seq, treated cells were harvested for RNA extraction using TRIzol. Reagent and genomic DNA was removed using DNase I (Takara). The sequencing library was constructed after high-quality RNA was quantified and then sequenced with the Illumina HiSeq X Ten (2 × 150 bp read length).

### Western blotting

Treated cells were lysed in RIPA buffer supplemented with phenylmethylsulphonyl fluoride (Beyotime Institute of Biotechnology, Haimen, China). The BCA method was used to assess protein concentration. Proteins were separated by 12.5% SDS-PAGE and transferred to PVDF membrane and immunoblotted with the following antibodies: anti-PLP2 (1:1000, ab180131, Abcam, Cambridge, MA, USA), anti-E-cadherin (1:1000, Abcam, ab231303), anti-Vimentin (Abcam, ab92547), anti-GAPDH (1:1000, Abcam, ab181603), and anti β-actin (1:1000, Abcam, ab8226). After rinsed with TBST, the membranes were incubated with HRP-conjugated secondary antibody (1:10000; cat. no. A0208; Beyotime Institute of Biotechnology), developed with an enhanced chemiluminescence reagent (GE Healthcare Bio-Sciences, Pittsburgh, PA, USA) and visualized by Image Lab software (Bio-Rad Laboratories, Hercules, CA, USA). The experiments were performed in triplicate.

### Cell immunofluorescence

UCEC cells were incubated with rabbit anti-human PLP2 antibody (1:200; ab180131, Abcam) overnight at 4 °C in a humid chamber. After washing three times with TBS (Beyotime, ST661), cells were incubated with dunkey anti-rabbit IgG H&L (Alexa Fluor® 488) preadsorbed antibody (1:400; 34206ES60, Yeasen, Shanghai, China). And cell nucleus was stained with 4′,6-diamidino-2-phenylindole (DAPI; Beyotime, C1006).

### Combined fluorescent *in situ* hybridization for miR-765 and immunofluorescent labeling for CD8

Fluorescent *in situ* hybridization (FISH) for miR-765 in UCEC tissues using the RiboTM Fluorescent *In situ* Hybridization Kit (RiboBio, China). After fixing in a 4% (wt/vol) paraformaldehyde solution, samples were rinsed in 1 × PBS, permeabilized in 1 × PBS with 0.5% (vol/vol) Triton X-100 (10 min), washed in 1 × PBS with 0.1% (vol/vol) Tween-20 (1 min). Hybridization was carried out using the miR-765 FISH Probe Mix (RiboBio, China) in a humidified chamber at 37 °C for overnight. After RNA FISH, cells were fixed again for 5 min in 2% formaldehyde and subjected to immunofluorescence with anti-CD8 primary antibody (1:200; ab217344, Abcam) and fluorescent secondary antibody were sequentially. Immediately after DAPI stain solution was added, images were taken with immunofluorescence microscope (Leica, TCS SP5II).

### Xenograft mouse model

UCEC cells (1 × 10^7^) stably expressing LV-miR-765-NC, LV-miR-765, LV-PLP2-NC, LV-si-PLP2, were subcutaneously injected into the left flank area of 4‑week‑old nude mice (n = 8 mice/group). Tumor volumes were measured everyday (0.5 × length × width^2^). Five weeks later, the mice were sacrificed and xenografts were assessed and weighed. Experiments on animals were approved by the Ethics Committee for Animal Experimentation of Obstetrics and Gynecology Hospital of Fudan University and strictly confirmed to the Institutional Guidelines for Use and Care of Laboratory Animals.

### *In vivo* imaging of tumor cell metastasis assay

Ishikawa cells were transfected with the reporter gene firefly luciferase (Ishikawa/luc) and treated *in vitro* either with control siRNA (NC) or PLP2 siRNA (siPLP2). 3 × 10^6^ Ishikawa/luc + NC, Ishikawa/luc + siPLP2 were transplanted intravenously into 4 weeks old female nude mice. After 6 weeks, endpoint measurements were performed using NightOwl LB 981 systems (Berthold Technologies GmbH & Co. KG, Bad Wildbad, Germany). Mice were anesthetized and intravenously injected with 150 μL luciferin (27 mg/mL, 200 mg/kg) (Biosynth, Staad, Switzerland). Measurements started 20 min after luciferin injection. For all images an exposition time of 5 min was chosen. After completion of *in vivo* imaging animals were sacrificed. The intensity of the bioluminescence signal was color coded and overlayed with bright field picture.

### Statistical analysis

All analyses were conducted by SPSS 25 (IBM). Between two groups, a paired or unpaired t test with or without Wilcoxon matched‐pairs signed‐rank test or a Mann‐Whitney U test was used according to the analysis of normal distribution and variance homogeneity. Among multiple groups, a one-way ANOVA test with or without a Kruskal-Wallis test according to the analysis of normal distribution and variance homogeneity. The data were presented as mean ± standard error (SEM) or median and quartile for normally distributed or other data. Log‑rank test was performed and Kaplan‑Meier survival curves were plotted. *P* < 0.05 was considered to indicate a statistically significant difference.

## Supplementary Material

Supplementary figures.

## Abbreviations

EECs: endometrial epithelial cells
EMT: epithelial-mesenchymal transition
ER: estrogen receptors
GPER-1: G-protein-coupled estrogen receptor-1
miRNAs: MicroRNAs
PLP2: Proteolipid protein 2
UCEC: Uterine corpus endometrial cancer
