# Supplementary material for: CD45RO-CD8+ T cell-derived exosomes restrict estrogen-driven endometrial cancer development via the ERβ/miR-765/PLP2/Notch axis
Source: Theranostics. 2021 Mar 11;11(11):5330–45. doi: 10.7150/thno.58337 (PMC8039953; doi:10.7150/thno.58337)
Supplement: Supplementary file 1 — Supplementary figures. [file thnov11p5330s1.pdf]

Supplemental information

Figure S1 Suppression of miR-765 level accelerates the proliferation and EMT of UCEC.

A Level of miR-765 after treated with miR-765 mimics in KLE by RT-qPCR. **B** KLE cell viability was measured by CCK-8 at 12 h, 24 h, 36 h, 48 h after treated with miR-765 mimics. **C** After treated with miR-765 mimics, KLE cell proliferation was determined with Ki67 by flow cytometry. **D** Tumor volume growth and of NC and LV-miR-765 KLE cells xenografts in mice were measured every day. **E** Survival curves of NC and LV-miR-765 KLE cells xenografts-bearing mice. **F** mRNA levels of EMT related markers (*COL1A, COL3, FN1, CDH1, CDH2, S100A, MMP2, MMP9, SNAIL, VIMENTIN, ZEB1, TJP1*) in NC and miR-765 mimics treated KLE cells by RT-qPCR. Data were presented as mean ± SEM and analyzed by t test or ANOVA. * $P < 0.05$, ** $P < 0.01$, *** $P < 0.001$, **** $P < 0.0001$, NS: no significance.

Figure S2 MiR-765 negatively regulates PLP2 expression of UCEC in a miRNAs cluster regulatory manner.

A The ratio of PLP2 to GAPDH was quantified after Ishikawa and KLE cells were treated with miR-765 mimics according to the images of western blotting in Figure 2D. **B** Potential transcription factors downstream miR-765 predicted by bioinformatics analysis. **C** Levels of target miRNAs (miR-3584-5p, miR-7-5p, miR-150-5p and miR-124-3p) after treated with miR-765 mimics in KLE cells by RT-qPCR. **D** Levels of PLP2 after treated with miR-3584-5p, miR-7-5p, miR-150-5p and miR-124-3p mimics in KLE cells by RT-qPCR. **E** Levels of target miRNAs (miR-3584-5p, miR-7-5p, miR-150-5p and miR-124-3p) were

assessed to evaluated the efficiency of miRNA inhibitors in KLE cell by RT-qPCR. **F** Levels of PLP2 after treated with miR-765 mimics and/or miRNA inhibitors in KLE cell by RT-qPCR. Data were presented as mean ± SEM and analyzed by t test or ANOVA. * P < 0.05, ** P < 0.01, *** P < 0.001, **** P < 0.0001, NS: no significance.

Figure S3 PLP2 promotes the EMT process and invasion of UCEC.

A-B RT-qPCR and western blotting were performed to evaluate the efficiency of siPLP2 respectively in Ishikawa and KLE. The ratio of PLP2 to GAPDH was quantified in (**C**). **D-E** The ratios of EMT related markers (Vimentin and E-cadherin) to GAPDH in NC or siPLP2 Ishikawa and KLE cells were quantified according to the images of western blotting in Figure.3C. * P < 0.05, ** P < 0.01, *** P < 0.001, **** P < 0.0001, NS: no significance.

Figure S4 PLP2 enhances the EMT process of UCEC by activation of Notch signaling.

A-B mRNA and protein levels of Notch-related molecules (Notch1, NID, Hes1) in NC and OE-PLP2 KLE cells by RT-qPCR. After NC or PLP2-OE KLE cells after treated with DART or not, levels of EMT related markers (E-cadherin and Vimentin) (**C**)**,** cell invasion **(D)** and cell viability (**E)** was detected. Data were presented as mean ± SEM and analyzed by t test or ANOVA. * P < 0.05, ** P < 0.01, *** P < 0.001, **** P < 0.0001, NS: no significance.

Figure S5 Estrogen regulates the miR-765/PLP2 level in UCEC through the ERβ.

A The ratio of PLP2 to GAPDH was quantified according to the images of western blotting in Figure. 5G. **B-C** The ratio of PLP2 to GAPDH was quantified according to the images of

western blotting in Figure. 5H-I. **D-E** The ratio of PLP2 to GAPDH was quantified according to the western blotting images of Figure. 5L-M. **F** Levels of ESR2 were assessed after Ishikawa and KLE cells were treated with Fulvestrant by RT-qPCR. **G** Levels of ESR1 were assessed after Ishikawa and KLE cells were treated with Fulvestrant by RT-qPCR. **H** Levels of miR-765 were assessed after Ishikawa and KLE cells were treated with Fulvestrant by RT-qPCR. **I-J** Levels of PLP2 were assessed after Ishikawa and KLE cells were treated with Fulvestrant by RT-qPCR and western blotting. The ratio of PLP2 to GAPDH was quantified in (**K**). **L** Levels of miR-765 were measured after NC or si-ERβ transfected Ishikawa and KLE cells were treated with Fulvestrant or not by RT-qPCR **M** Levels of PLP2 were assessed after NC or si-ERβ transfected Ishikawa ~~and KLE~~ cells were treated with Fulvestrant or not by immunofluorescence. Data were presented as mean ± SEM and analyzed by t test or ANOVA. * $P < 0.05$, ** $P < 0.01$, *** $P < 0.001$, **** $P < 0.0001$, NS: no significance.

Figure S6 CD45RO^{-}CD8^{+} T cell-derived exosomal miR-765 partly suppressed PLP2 in UCEC cells.

A Sorting strategy of CD45RO^{+}CD8^{+} and CD45RO^{-}CD8^{+} T cells by flow cytometry. **B** Histogram showing the particle diameter (nm) of the exosomes. **C** Exosome markers, CD63, TSG101, and GRP78, were assayed in exosomes and lysate using western blotting. The ratio of CD63/TSG101/GRP78 to PSMA was quantified in **(D-F)**. **G** Levels of miR-765 in KLE were tested after treated with CD45RO^{-}CD8^{+}T cells exosomes by RT-qPCR. **H** Levels of PLP2 in KLE were tested after treated with CD45RO^{-}CD8^{+} T cell-derived exosomes by RT-qPCR. **I** The ratio of PLP2 to GAPDH was quantified according to images of western

blotting in Figure. 6G. **J-K** mRNA and protein levels of PLP2 in KLE were tested after treated with $CD45RO^{-}CD8^{+}$ T cell-derived exosomes and/or GW4869 by RT-qPCR and western blotting. Data were presented as mean ± SEM and analyzed by t test or ANOVA. * P < 0.05, ** P < 0.01, *** P < 0.001, **** P < 0.0001, NS: no significance.

Figure S7 Exosomes originated from $CD45RO^{-}CD8^{+}$ T cell partly suppress UCEC development in a miR-765/PLP2-dependent manner.

After KLE cells were treated with estrogen and/or $CD45RO^{-}CD8^{+}$T cell-derived exosomes for 24h, **A** Levels of miR-765 were tested by RT-qPCR. **B-C** Levels of PLP2 were tested by RT-qPCR and western blotting. **D** Cell viability was measured by CCK-8 at 12 h, 24 h, 36 h, 48 h. **E** Cell proliferation was determined with Ki67 by flow cytometry. **F** Levels of EMT related markers (Vimentin and E-cadherin) were measured by western blotting and were quantified in **(G-H),** respectively. **I** Cell invasion was detected by transwell assays. Scale bars: 100 μm. **J** Ultimate tumor volume of ~~Ishikawa~~ cells xenografts in mice were measured. **K** Survival curves of KLE cells xenografts-bearing mice was plotted. Data were presented as mean ± SEM and analyzed by t test or ANOVA. * P < 0.05, ** P < 0.01, *** P < 0.001, **** P < 0.0001, NS: no significance.

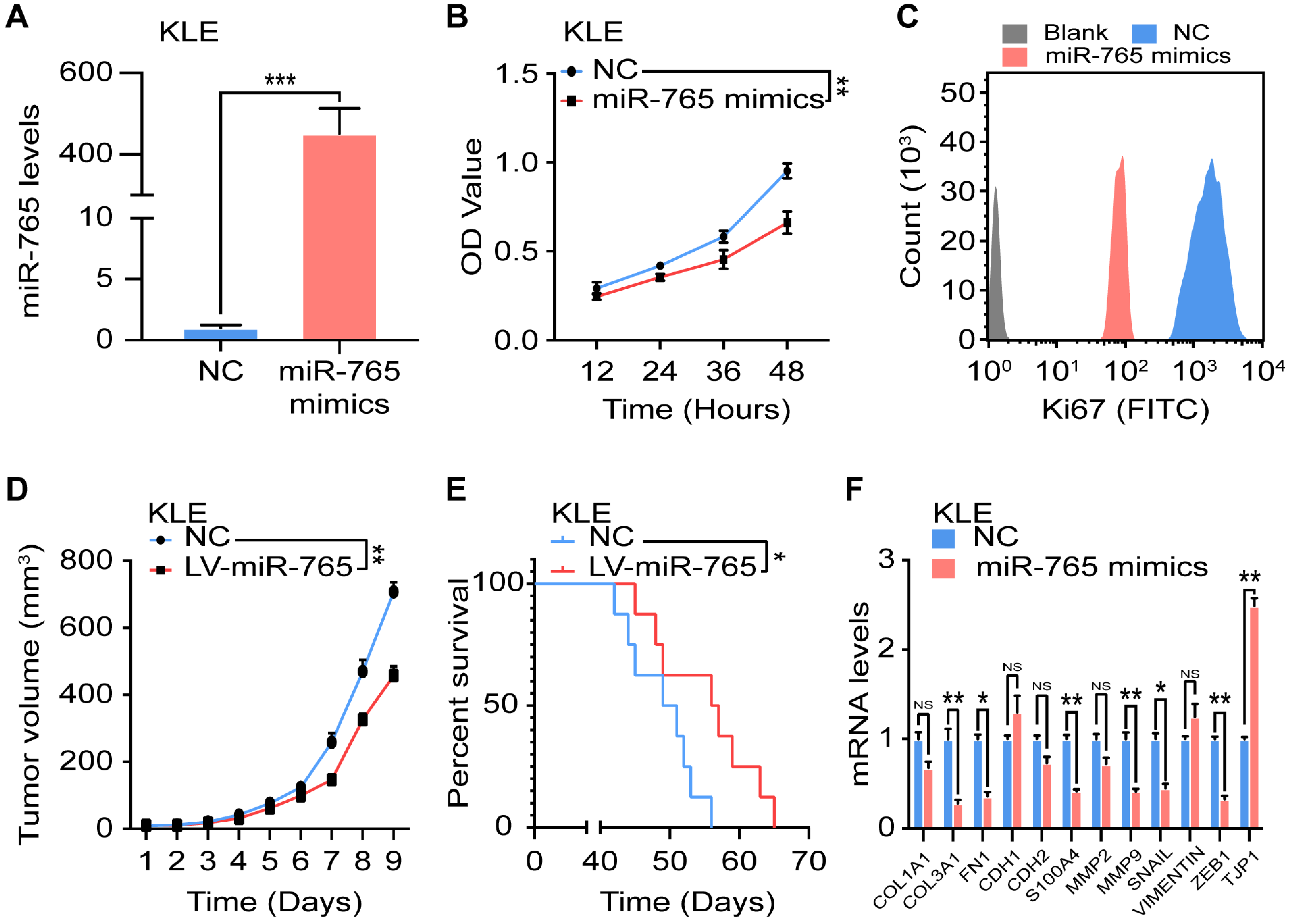

A
KLE
miR-765 levels
600
400
10
5
0
***
NC
miR-765 mimics
B
KLE
OD Value
1.5
1.0
0.5
0.0
NC
miR-765 mimics
**
12
24
36
48
Time (Hours)
C
Blank
NC
miR-765 mimics
Count (10³)
50
40
30
20
10
0
10⁰
10¹
10²
10³
10⁴
Ki67 (FITC)
D
KLE
NC
LV-miR-765
**
Tumor volume (mm³)
800
600
400
200
0
1
2
3
4
5
6
7
8
9
Time (Days)
E
KLE
NC
LV-miR-765
*
Percent survival
100
50
0
0
40
50
60
70
Time (Days)
F
KLE
NC
miR-765 mimics
mRNA levels
3
2
1
0
NS
**
*
NS
NS
**
NS
**
*
NS
**
**
COL1A1
COL3A1
FN1
CDH1
CDH2
S100A4
MMP2
MMP9
SNAIL
VIMENTIN
ZEB1
TJP1

Figure S1

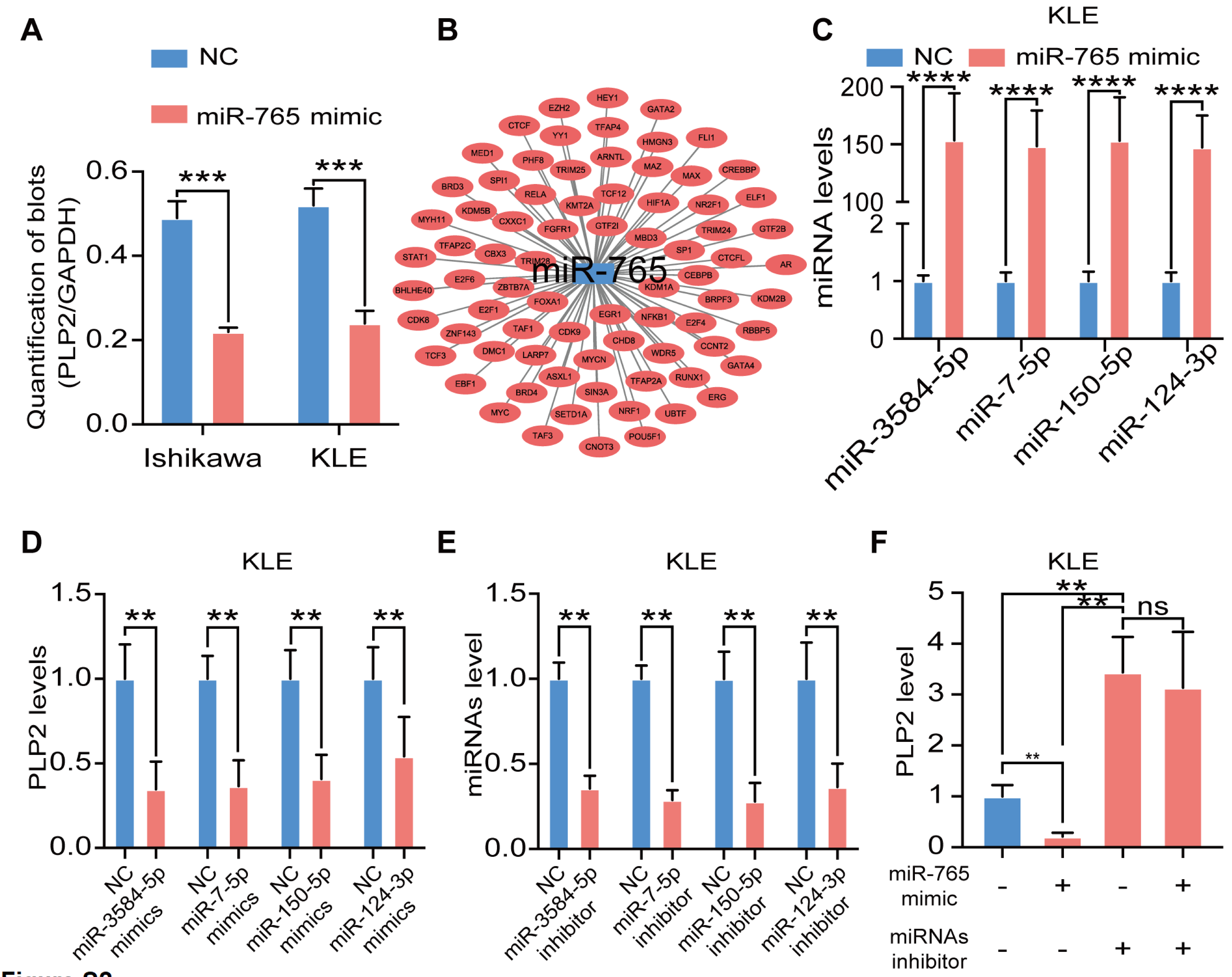
A
NC
miR-765 mimic
Quantification of blots (PLP2/GAPDH)
0.0
0.2
0.4
0.6
***
***
Ishikawa
KLE
B
miR-765
HEY1
EZH2
GATA2
CTCF
TFAP4
YY1
FLI1
HMGN3
MED1
ARNTL
PHF8
MAZ
CREBBP
TRIM25
MAX
BRD3
SPI1
RELA
TCF12
ELF1
KMT2A
HIF1A
NR2F1
MYH11
KDM5B
CXXC1
GTF2I
FGFR1
TRIM24
GTF2B
MBD3
TFAP2C
CBX3
SP1
STAT1
TRIM28
CTCFL
AR
CEBPB
E2F6
ZBTB7A
KDM1A
BHLHE40
BRPF3
KDM2B
FOXA1
E2F1
EGR1
NFKB1
CDK8
E2F4
TAF1
CDK9
RBBP5
ZNF143
CHD8
CCNT2
DMC1
WDR5
TCF3
LARP7
MYCN
GATA4
ASXL1
RUNX1
EBF1
TFAP2A
SIN3A
BRD4
ERG
MYC
NRF1
SETD1A
UBTF
TAF3
POU5F1
CNOT3
C
KLE
NC
miR-765 mimic
miRNA levels
0
1
2
100
150
200
****
****
****
****
miR-3584-5p
miR-7-5p
miR-150-5p
miR-124-3p
D
KLE
PLP2 levels
0.0
0.5
1.0
1.5
**
**
**
**
NC
miR-3584-5p mimics
NC
miR-7-5p mimics
NC
miR-150-5p mimics
NC
miR-124-3p mimics
E
KLE
miRNAs level
0.0
0.5
1.0
1.5
**
**
**
**
NC
miR-3584-5p inhibitor
NC
miR-7-5p inhibitor
NC
miR-150-5p inhibitor
NC
miR-124-3p inhibitor
F
KLE
PLP2 level
0
1
2
3
4
5
**
**
ns
**
miR-765 mimic
- + - +
miRNAs inhibitor
- - + +

Figure S2

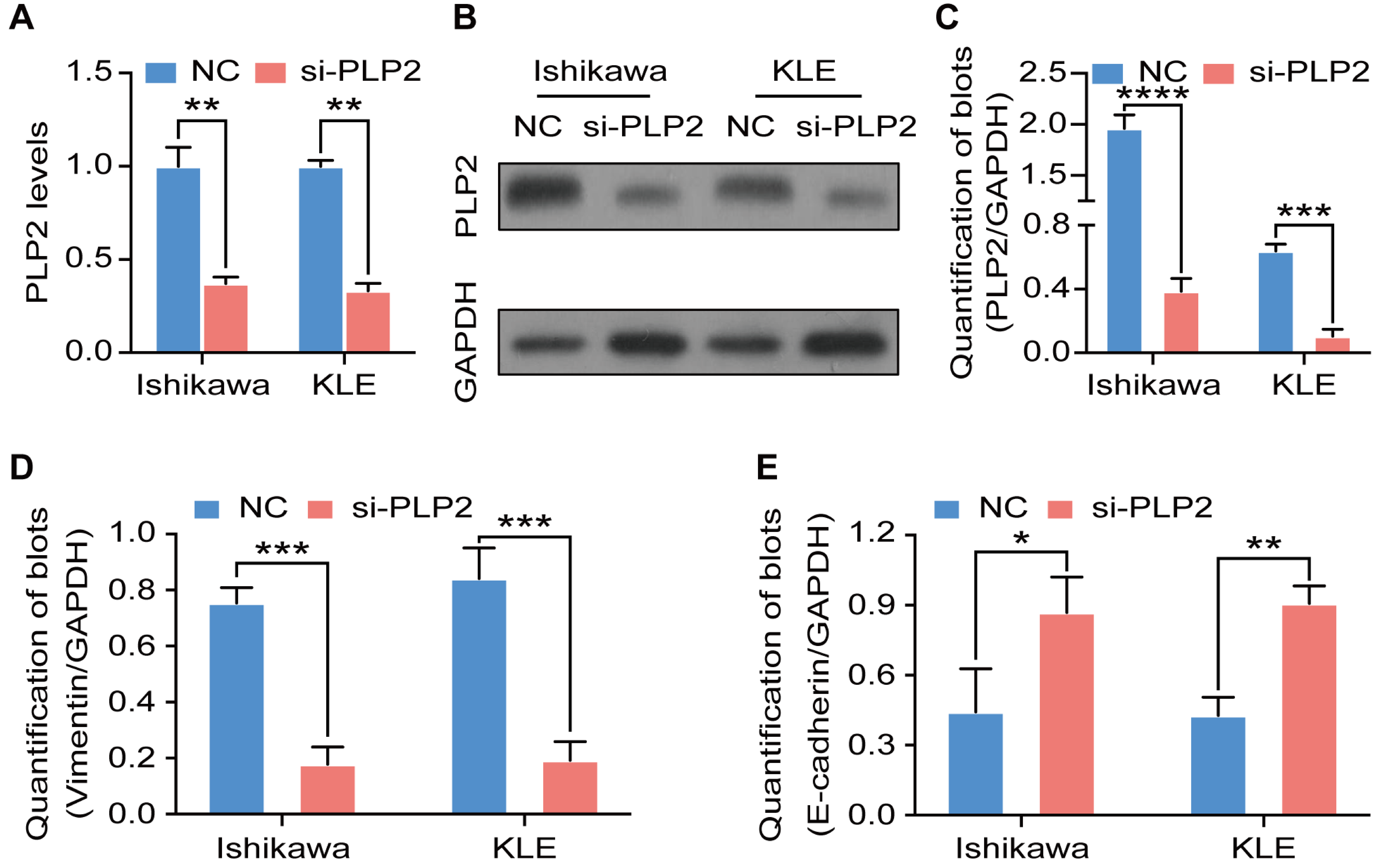

A
B
C
D
E
NC
si-PLP2
PLP2 levels
Ishikawa
KLE
PLP2
GAPDH
Quantification of blots (PLP2/GAPDH)
Quantification of blots (Vimentin/GAPDH)
Quantification of blots (E-cadherin/GAPDH)

Figure S3

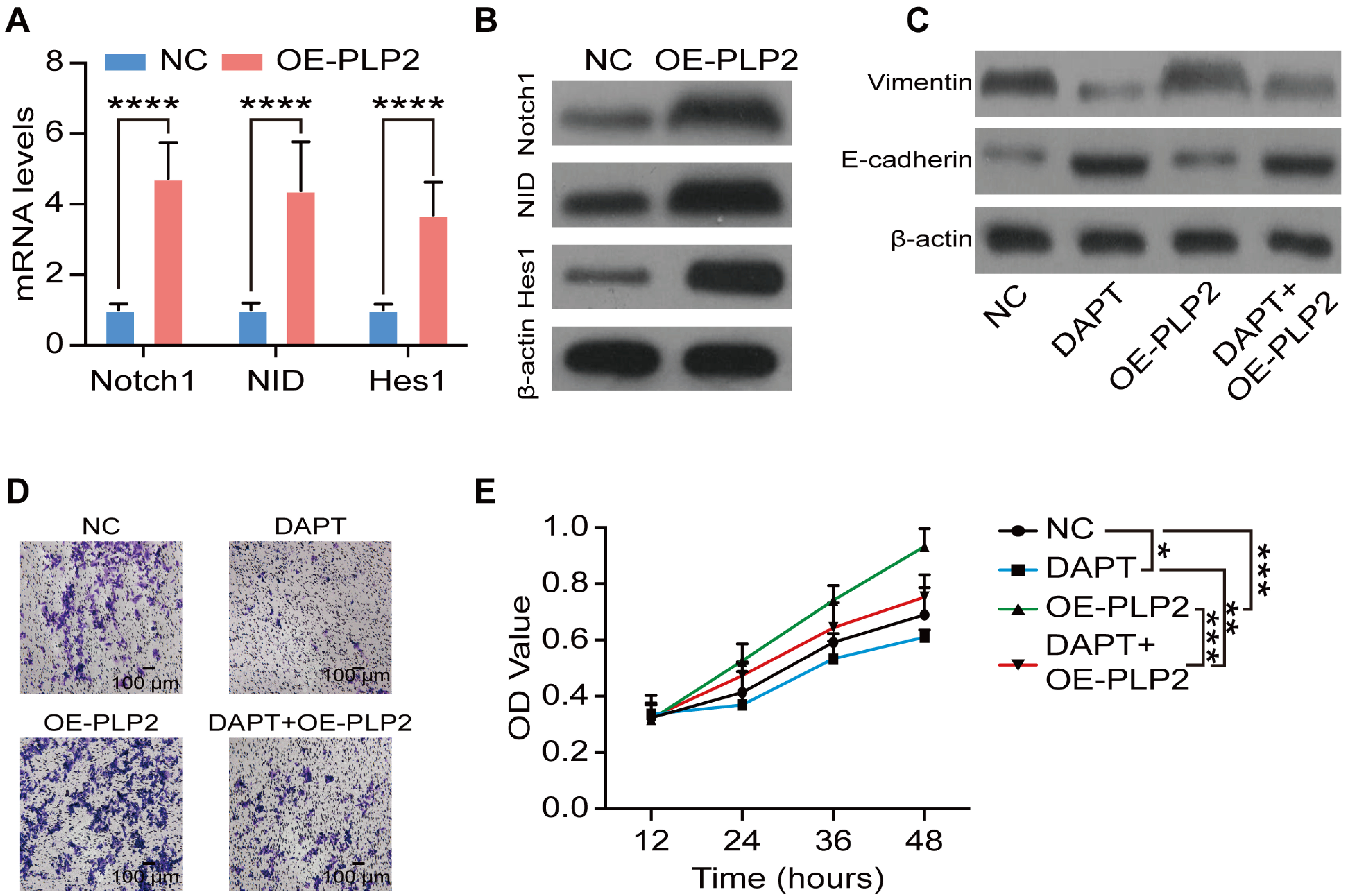

A
NC
OE-PLP2
mRNA levels
8
6
4
2
0
****
****
****
Notch1
NID
Hes1
B
NC
OE-PLP2
Notch1
NID
Hes1
β-actin
C
Vimentin
E-cadherin
β-actin
NC
DAPT
OE-PLP2
DAPT+
OE-PLP2
D
NC
DAPT
OE-PLP2
DAPT+OE-PLP2
100 μm
100 μm
100 μm
100 μm
E
OD Value
1.0
0.8
0.6
0.4
0.2
0.0
12
24
36
48
Time (hours)
NC
DAPT
OE-PLP2
DAPT+
OE-PLP2
*
***
**
***

Figure S4

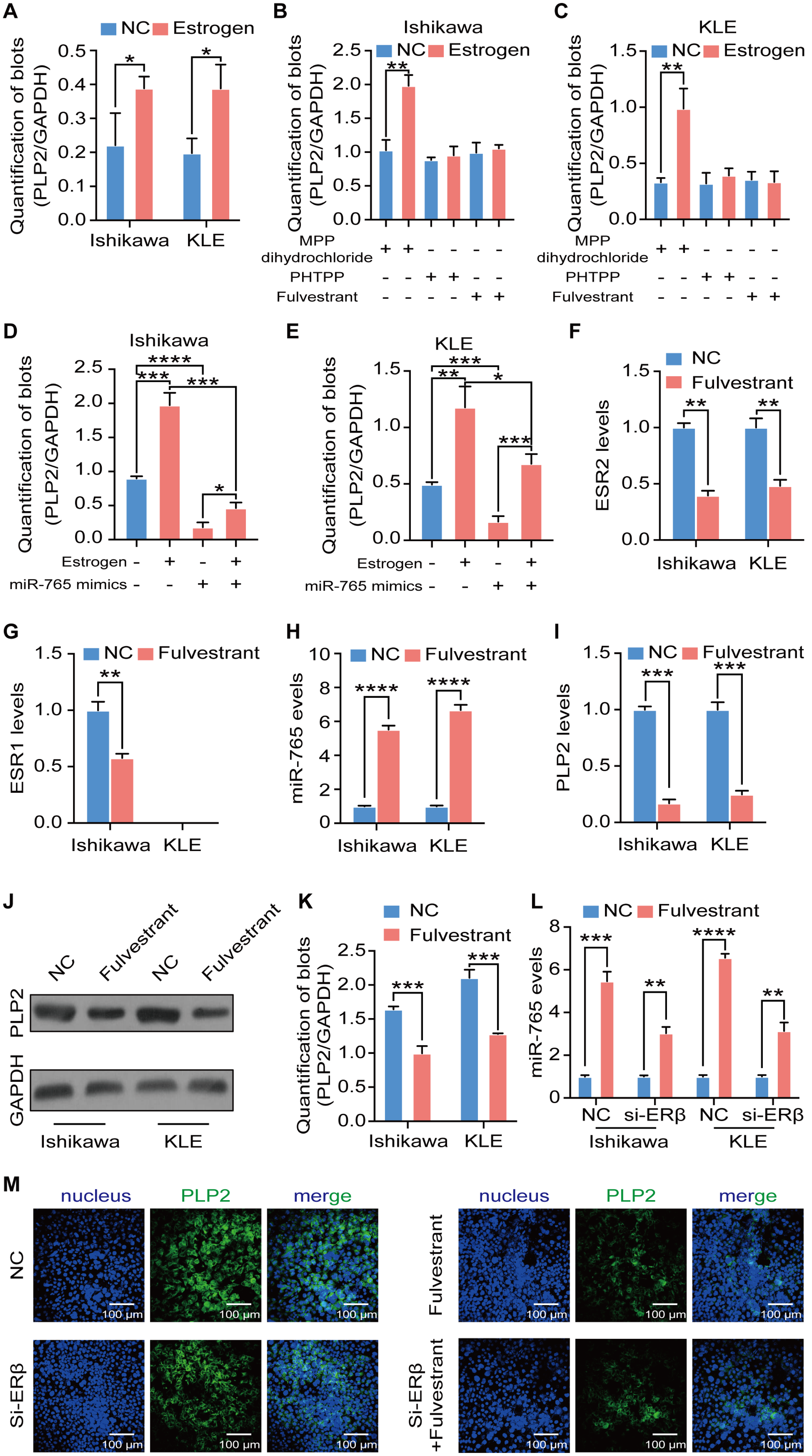
A
NC
Estrogen
Quantification of blots (PLP2/GAPDH)
Ishikawa
KLE
B
Ishikawa
MPP dihydrochloride
PHTPP
Fulvestrant
C
KLE
D
E
Estrogen
miR-765 mimics
F
ESR2 levels
Fulvestrant
G
ESR1 levels
H
miR-765 evels
I
PLP2 levels
J
PLP2
GAPDH
K
L
si-ERβ
M
nucleus
PLP2
merge
NC
Si-ERβ
Si-ERβ +Fulvestrant
100 μm

Figure S5

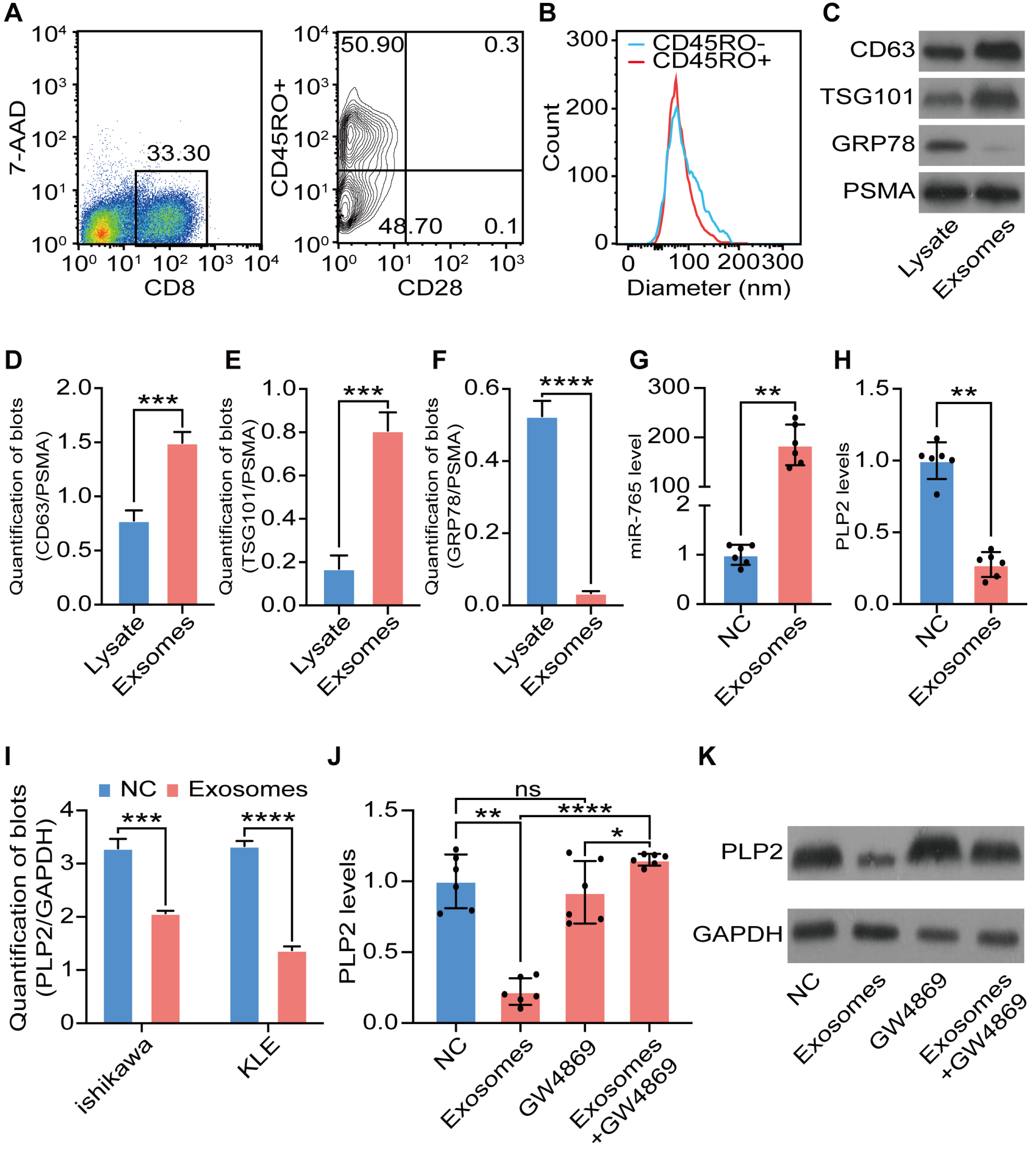

A
B
C
D
E
F
G
H
I
J
K
7-AAD
CD8
33.30
CD45RO+
CD28
50.90
0.3
48.70
0.1
Count
Diameter (nm)
CD45RO-
CD45RO+
CD63
TSG101
GRP78
PSMA
Lysate
Exsomes
Quantification of blots (CD63/PSMA)
Quantification of blots (TSG101/PSMA)
Quantification of blots (GRP78/PSMA)
miR-765 level
PLP2 levels
NC
Exosomes
Quantification of blots (PLP2/GAPDH)
ishikawa
KLE
ns
GW4869
Exosomes +GW4869
PLP2
GAPDH

Figure S6

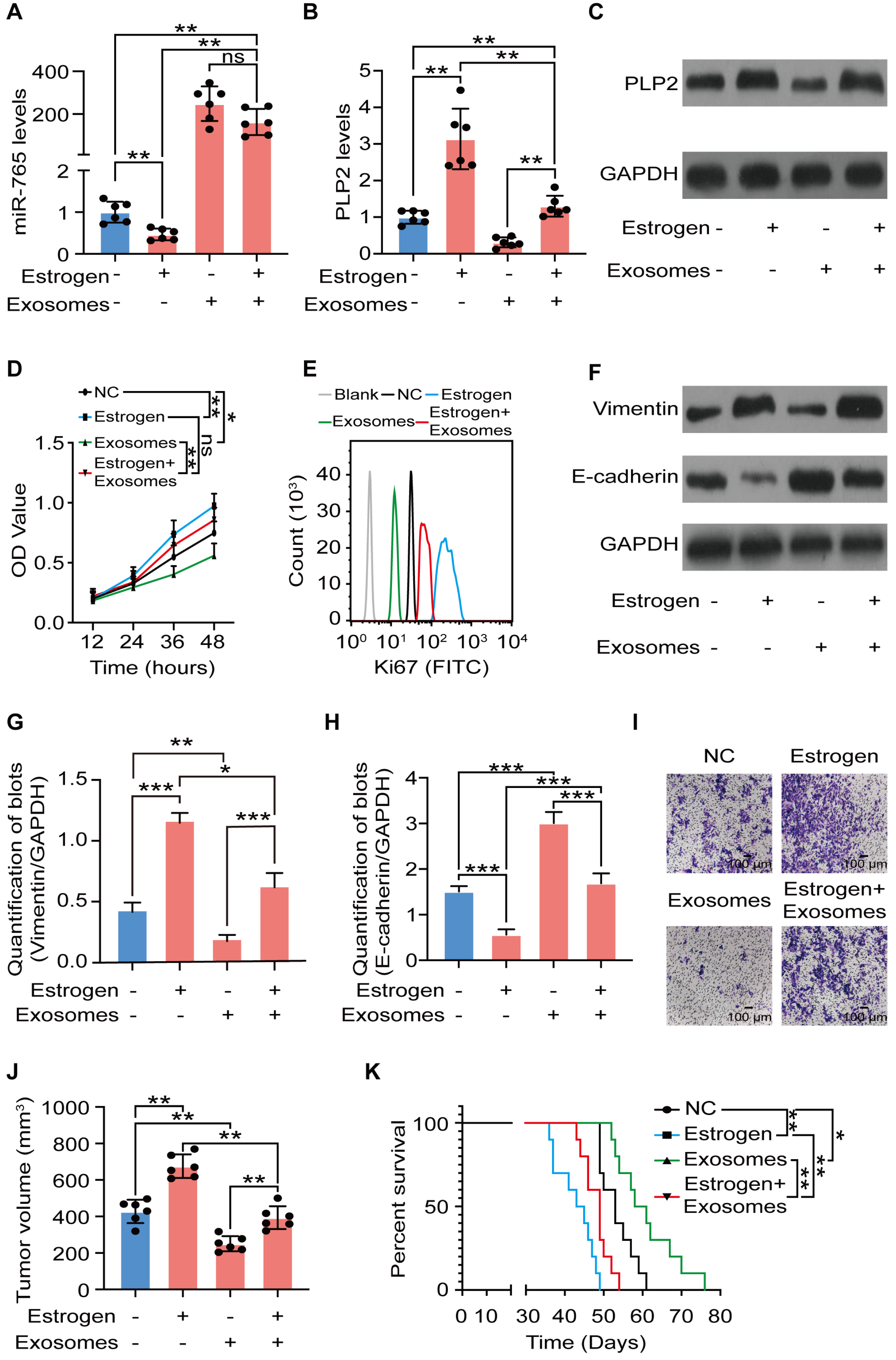
A
miR-765 levels
Estrogen - + - +
Exosomes - - + +
ns
B
PLP2 levels
C
PLP2
GAPDH
D
NC
Estrogen
Exosomes
Estrogen+ Exosomes
OD Value
Time (hours)
E
Blank NC Estrogen
Exosomes Estrogen+ Exosomes
Count (10³)
Ki67 (FITC)
F
Vimentin
E-cadherin
GAPDH
G
Quantification of blots (Vimentin/GAPDH)
H
Quantification of blots (E-cadherin/GAPDH)
I
NC
Estrogen
Exosomes
Estrogen+ Exosomes
100 μm
J
Tumor volume (mm³)
K
Percent survival
Time (Days)

Figure S7
